# Genomic wide association study and selective sweep analysis identify genes associated with improved yield under drought in Turkish winter wheat germplasm

**DOI:** 10.1038/s41598-024-57469-1

**Published:** 2024-04-10

**Authors:** Deepmala Sehgal, Nagenahalli Dharmegowda Rathan, Fatih Özdemir, Mesut Keser, Beyhan Akin, Abdelfattah A. Dababat, Emrah Koc, Susanne Dreisigacker, Alexey Morgounov

**Affiliations:** 1 International Maize and Wheat Improvement Center (CIMMYT), Km. 45, Carretera Mex-Veracruz, El Batan, CP 56237 Veracruz, Mexico; 2 Syngenta, Jealott’s Hill International Research Centre, Bracknell, Berkshire, RG42 6EY UK; 3 Corteva Agriscience, Hyderabad, Telangana India; 4 Bahri Dagdas International Agricultural Research Institute, Konya, Turkey; 5 International Center for Agricultural Research in Dry Areas (ICARDA), Ankara, Turkey; 6 International Maize and Wheat Improvement Center (CIMMYT), Ankara, Turkey; 7 Scientific Production Center of Grain, Shortandy, Astana reg., 010000 Kazakhstan

**Keywords:** Agricultural genetics, Plant stress responses

## Abstract

A panel comprising of 84 Turkish winter wheat landraces (LR) and 73 modern varieties (MV) was analyzed with genome wide association study (GWAS) to identify genes/genomic regions associated with increased yield under favorable and drought conditions. In addition, selective sweep analysis was conducted to detect signatures of selection in the winter wheat genome driving the differentiation between LR and MV, to gather an understanding of genomic regions linked to adaptation and yield improvement. The panel was genotyped with 25 K wheat SNP array and phenotyped for agronomic traits for two growing seasons (2018 and 2019) in Konya, Turkey. Year 2018 was treated as drought environment due to very low precipitation prior to heading whereas year 2019 was considered as a favorable season. GWAS conducted with SNPs and haplotype blocks using mixed linear model identified 18 genomic regions in the vicinities of known genes i.e., *TaERF3-3A, TaERF3-3B*, *DEP1-5A, FRIZZY PANICLE-2D, TaSnRK23-1A, TaAGL6-A*, *TaARF12-2A, TaARF12-2B, WAPO1, TaSPL16-7D, TaTGW6-A1, KAT-2B, TaOGT1, TaSPL21-6B, TaSBEIb, trs1/WFZP-A, TaCwi-A1-2A* and *TaPIN1-7A* associated with grain yield (GY) and yield related traits. Haplotype-based GWAS identified five haplotype blocks (H1A-42, H2A-71, H4A-48, H7B-123 and H7B-124), with the favorable haplotypes showing a yield increase of > 700 kg/ha in the drought season. SNP-based GWAS, detected only one larger effect genomic region on chromosome 7B, in common with haplotype-based GWAS. On an average, the percentage variation (PV) explained by haplotypes was 8.0% higher than PV explained by SNPs for all the investigated traits. Selective sweep analysis detected 39 signatures of selection between LR and MV of which 15 were within proximity of known functional genes controlling flowering (*PRR-A1*, *PPR-D1*, *TaHd1-6B*), GY and GY components (*TaSus2*-2B, *TaGS2-B1*, *AG1-1A/WAG1-1A*, *DUO-A1*, *DUO-B1, AG2-3A/WAG2-3A*, *TaLAX1, TaSnRK210-4A, FBP, TaLAX1*, *TaPIL1* and *AP3-1-7A/WPA3-7A*) and 10 regions underlying various transcription factors and regulatory genes. The study outcomes contribute to utilization of LR in breeding winter wheat.

## Introduction

Wheat is a major staple cereal, providing one-fifth of the calories and protein for four billion people globally^1^. Turkey is the eighth largest wheat producer worldwide and a leading exporter cultivating > 8 million ha^2^. Wheat production in Turkey is therefore important for global food security. Climate change scenarios have indicated that heat and drought stress will have profound effects on Turkey’s wheat production in coming decades^3,4^. Latest crop models project that the winter wheat zone of the country will suffer great losses in yield as compared to the spring wheat zone due to drought and heat (25–29%) yield reductions in winter wheat and 15–16% in spring wheat, respectively^5^. There is an urgent need to explore new approaches to develop climate resilient wheat varieties that can adapt to drought and heat stress environments.

In 2011–2014, International Winter Wheat Improvement Network (IWWIP) based in Turkey, collected wheat landraces (LR), which were evaluated, and superior landraces multiplied for seed deliveries to farming communities assuring their continuous cultivation and use in breeding^6^. The LR collection was sampled from four provinces in Turkey, two provinces in Iran and Afghanistan. Thereafter, 84 LR were delivered to the gene banks of the three countries to establish a common field trial along with 73 IWWIP modern germplasm and varieties (MV). This important germplasm set, named ‘International Landrace Exchange Set’ was evaluated for yield and yield components across Afghanistan, Iran and Turkey^6^. The trial results demonstrated that LR were highly adaptable to diverse agro-ecological conditions in all three countries.

Genome-wide association study (GWAS) has been increasingly utilized in wheat to untangle the genetic architecture of complex agronomic traits including yield and yield components under favorable and abiotic stress conditions^7–17^. However, most studies have used SNP-based GWAS. SNP markers are bi-allelic therefore less informative than multi-allelic markers. Additionally, in SNP-based GWAS multiple SNPs in high linkage disequilibrium (LD) are commonly associated with the same quantitative trait loci (QTL), resulting in overestimation of QTL effects. Haplotypes are constructed by combining multiple SNPs in high LD and haplotype-based GWAS overcomes most of the limitations associated with using single SNPs in GWAS, resulting in better statistical significance^9,12,18–21^.

The increased understanding of genetic loci that control complex traits and those that underwent selection during crop improvement (referred to as selective sweeps or signatures of selection) is important to design efficient breeding strategies. A combination of GWAS and selective sweep analysis is emerging as a leading approach to identify such new genomic targets^14,22,23^. Additionally, it allows to evaluate the role of selection in shaping the quantitative genetic variation at various levels^24^. In this study, we characterized the International Landrace Exchange Set to (i) identify the genetic diversity and population structure in this set, (ii) conduct GWAS using SNP and haplotype-based GWAS to detect genomic regions leading to improved drought resilience, and (iii) identify genome regions that were directionally selected between LR and MV and link these regions to known genes related to wheat adaptation and improvement.

## Methods

### Germplasm panel

The LR subset in the International Landrace Exchange Set comprised of 84 wheat landraces; 45 entries from Turkey, 20 from Afghanistan and 19 from Iran. The MV subset comprised of 73 entries developed by IWWIP in Turkey; 32 were bred for semiarid environments and 41 for irrigated environments. The detail list of entries is in supplementary Table S1 and described by^6^.

### Phenotyping, genotyping and population structure

Phenotyping was conducted on 6 m2 plots at the Bahri Dagdas International Agricultural Research Institute in Konya, Turkey for two years (2018 and 2019). An alpha-lattice experimental design was used with two replicates. The weather conditions in Konya in 2018 were characterized by lack of moisture prior to heading resulting in drought conditions. In 2019, the precipitation was sufficient and grain yield exceeded 4 t/ha without applying any additional irrigation. The 2018 year was treated as a drought season and 2019 as a favorable season. Experimental data were recorded on grain yield (GY), spike length (SL), spike number (SN), number of spikelets per spike (NSS), harvest index (HI) and thousand grain weight (TGW) as described in^6^. The statistical analysis of the phenotypic data, including estimation of best linear unbiased predictors (BLUP) and broad-sense heritability, was done in Meta R^25^ (Vargas et al. 2013). Broad-sense-heritability in Meta R was estimated using the formula H^2^ = V_*g*_*/(*V_*g*_ + V_*err*_*/*r*)*, where V_*g*_ is the genotypic variance, V_*err*_ is the error variance, and r is the number of replications. The ANOVA was done with R package *lme4* using a linear mixed effect model in which replications were treated as fixed effect and entries as random effect. The correlations between traits were calculated using R packages *ggplot2*, *GGally* and *rlang*.

The germplasm set was genotyped using a high-density Illumina Infinium 25 K wheat SNP array (TraitGenetics GmbH, Gatersleben, Germany). After removing markers with missing data > 30% and minor allele frequency < 5%, 15,208 SNPs were used in the analyses. The polymorphic information content (PIC) and nucleotide diversity parameter (π) were calculated to estimate genetic diversity in the panel. PIC was calculated using PowerMarker version 3.25^26^ while π was calculated in TASSEL version 5.2.79^27^.

Principal component analysis (PCA) was conducted using the R package ‘stats’ and ‘rgl’. Coefficient of correlation *r*^*2*^ among markers was calculated to estimate LD among all pairwise comparison of markers in TASSEL version 5.2.79 and the values were plotted against genetic distance (bp) in R Studio using an in-house script. The pattern of LD decay was determined as the distance where LD values reduced to half of their maximum value.

### SNP and haplotype-based GWAS

Genome-wide haplotypes were constructed based on the linkage disequilibrium (LD) parameter D’ using the modified R script^28^. The details of the parameters used were described by^29,30^. Haplotype-based GWAS was conducted using Plink version 1.07 with default parameters^31^. SNP-based GWAS was conducted in GAPIT Version 3.0^32^. A mixed linear model was applied with the first three principal components as fixed variate and kinship as a random variate. SNPs and haplotype blocks were declared significant at *p* < 0.001. Box plots were generated to show the allelic effects of the associated markers and haplotype blocks using the PAST statistical program version 1.93^33^.

### Analyses of selective sweeps

To identify genomic regions under selection, we used two independent methods; EigenGWAS^34^ and Wright’s Fst statistic^35^. EigenGWAS (Genome-wide association study with eigenvector decomposition) is a GWAS, however, phenotypic data are replaced by individual-level eigenvectors derived from the genotypic data. EigenGWAS was conducted using R-based GEAR software (https://github.com/gc5k/GEAR). To control the genetic drift component, the method generates a genomic control factor (λ*GC*) and corrects the *p-*value. We used the corrected *p*‐value, called *P*GC (*p* value with a genomic control factor^36^, for detecting the loci under selection. One of the EigenGWAS outputs is called strength of selection, which is defined as the ratio between *F*_st_ of a locus and the average *Fst* of the population under study^37^. Wright’s F statistics of all individual 15,208 loci was calculated using ‘hierfstat’ package in R environment. A locus was declared under selection if it showed Fst > average Fst of the panel.

### Candidate gene analysis

To search for putative candidate genes in the proximity of any significant GWAS output, Basic Local Alignment Search Tool (BLAST) in the EnsemblPlant database (https://plants.ensembl.org/index.html) was used. The co-expression patterns and gene network analysis were investigated in the Wheat Expression database (http://www.wheat-expression.com/). Potential links to phenotypes were determined using Knetminer integrated in the Wheat Expression database.

## Results

### Phenotypic variation in LR and MV

Year 2018 growing season was treated as the drought environment due to very low precipitation prior to heading, whereas year 2019 growing season was considered as the favorable environment. The metrological data is provided in Table S2, which clearly supports the drier conditions in 2018 growing seasons as compared to 2019. The total rainfall during 2018 (149.6 mm) growing season was 27.7% lower than that during 2019 (206.8 mm) growing season. Analysis of variance (ANOVA) revealed that all traits showed significant variation in the panel (Table S3). The effect of environment was significant on all traits except SL. In addition, significant genotype x environment interaction was observed for all traits. Broad sense heritability (*H*^*2*^) estimates were higher in the drought season for GY, SL and SN, whereas these were higher for HI and TGW in the favorable season (Table S3). For NSS, *H*^*2*^ estimates were similar in both seasons. Across seasons, TGW had the highest heritability of 0.73 whereas SL and NSS showed moderate heritability values of 0.62 and 0.65, respectively. The remaining traits GY, SN and HI showed low heritability values (*H*^*2*^ < 0.50) across seasons.

**Figure 1:**
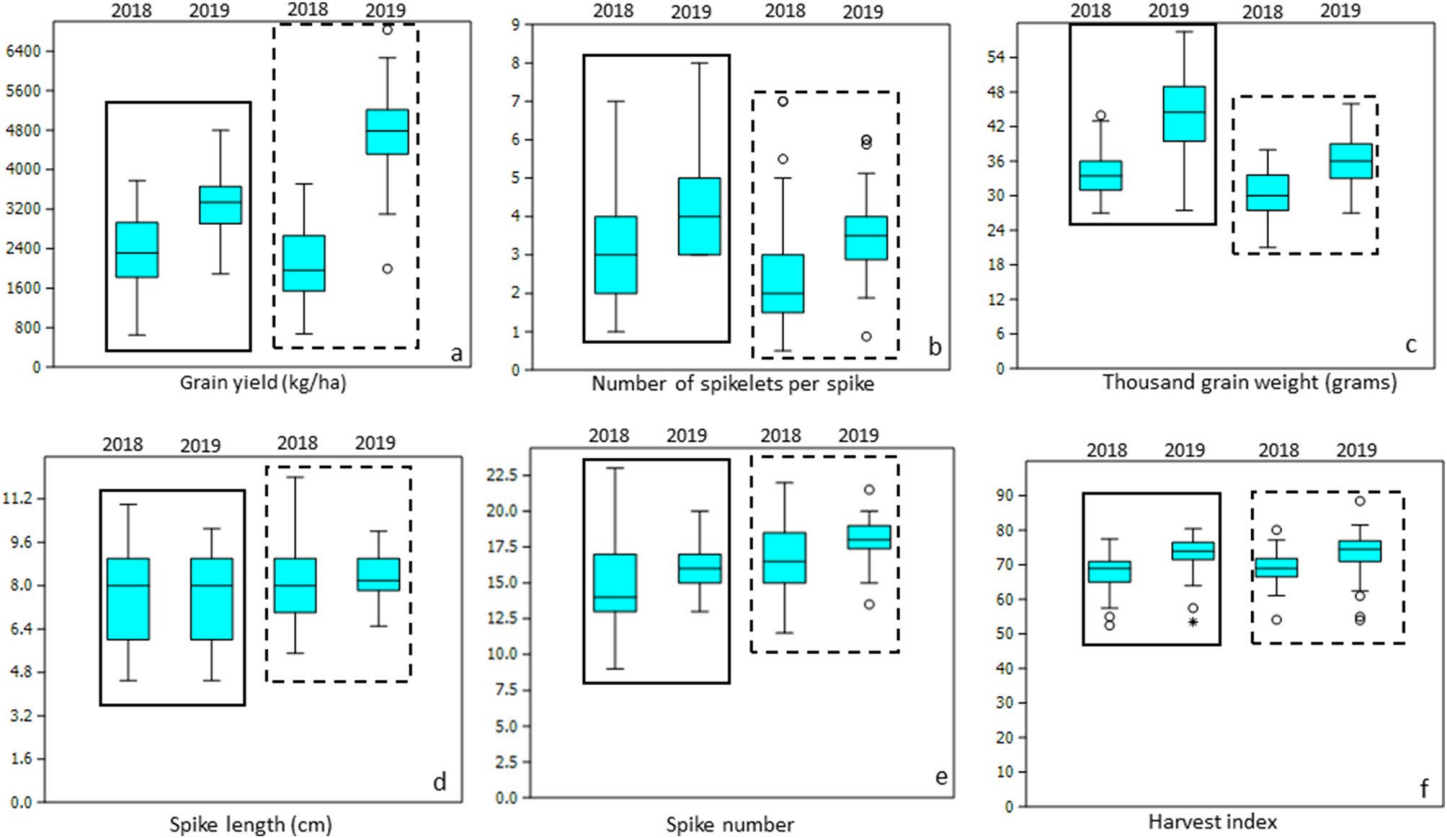
Rate of reduction in grain yield in kg/ha, p < 0.01 (**a**) and yield parameters (**b** number of spikelets per spike, p < 0.001; (**c**) thousand grain weight in g, p < 0.01, (**d**) spike length in cm, p < 0.05, (**e**) spike number, p < 0.01and (**f**) Harvest index, p < 0.05 in drought (2018) and favorable (2019) seasons. The solid and dotted boxes represent reductions LR and MV, respectively.

A detailed description of morphological diversity and descriptive statistics of the panel has been published recently^6^. To avoid repetition, we have elaborated here effects of drought on GY and yield components in LR and MV. The average GY was 3292 and 4786 kg/ha in the favorable season and 2378 and 2073 kg/ha in the drought season in LR and MV, respectively. This shows that reduction in GY was more severe in MV (56.6%) as compared to LR (27.7%) under drought conditions (Fig. 1a). NSS and TGW also showed significant reductions in the drought season in both LR and MV. The reduction in NSS was up to 33.3 and 30.9% whereas in TGW the reductions were up to 23.4 and 16.7% in LR and MV, respectively (Fig. 1b,c). For the remaining traits, reductions were moderate to very low (Fig. 1d–f). For example, SL was least affected by drought (Fig. 1d) in both LR and MV, while SN (Fig. 1e) and HI (Fig. 1f) displayed moderate reductions of 8.1 and 8.4% and 7.5 and 6.7% in LR and MV, respectively.

The correlations were estimated between traits for the two groups (LR and MV) and two seasons i.e. drought (2018) and favorable (2019). In general, the correlation between traits were higher in the drought season of (Fig. S1a,b) than in the favorable season of 2019 (Fig. S1c,d). In the favorable season, except for HI (r = 0.236, *p* < 0.05) in LR, correlations of all traits with GY were insignificant in both LR and MV. In the drought season, the correlation of three traits (SL, NSS and HI) became stronger and significant with GY in LR (SL and GY, r = 0.265 at *p* < 0.05; NSS and GY, r = -0.33 at *p* < 0.01; HI and GY, r = 0.367 at *p* < 0.001). In MV too, the correlation of all three spike traits (SL, SN and NSS) became stronger with GY in the drought season (SL and GY, r = 0.463 at *p* < 0.001; SN and GY, r = 0.391 at *p* < 0.001; NSS and GY, r = 0.432 at *p* < 0.001).

### Distribution of SNPs and haplotype blocks, population structure and LD decay

A total of 15,208 filtered SNPs was used, after filtering for 30% missing data and minor allele frequency ≥ 0.05, for all subsequent analysis. The distribution of SNPs showed maximum number of SNPs on chromosome 2B (1263) followed by chromosomes 3B (1196), 7A (1195) and 5B (1191). Chromosome 4D showed the least number of SNPs. Based on the linkage disequilibrium approach^28^, a total of 2568 haplotype blocks were constructed from 15,208 SNPs that covered a total genome length of 14,050 Mb (Table S4). The highest number of haplotype blocks were obtained on chromosome 5B (225) followed by chromosomes 3B (209) and 2B (205) (Table S4, Fig. S2).

**Figure 2:**
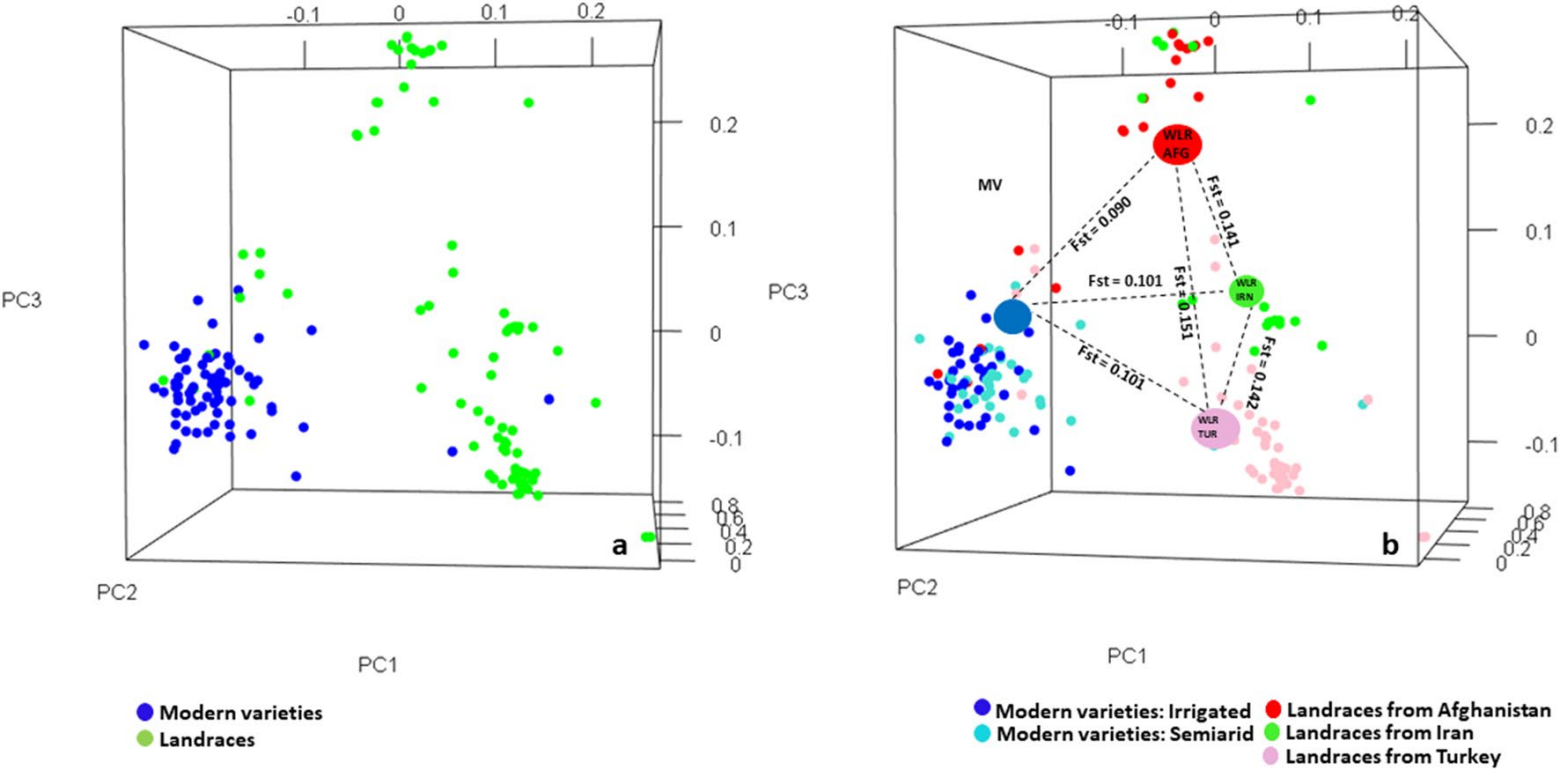
Three dimensional PCA plots showing two broad groups of LR and MV (**a**) and three subgroups of LR based on geographic origins and one group of MV (**b**). Pairwise Fst among different groups are shown in part (**b**) of the figure.

The population structure was determined by 3-dimensional PCA that revealed a clear distinction between LR and MV (Fig. 2a). Three subgroups were evident in the PCA plot. Subgroup 1 was formed by all modern varieties regardless of the fact whether these were bred for irrigated or semiarid environments while subgroups 2 and 3 were formed by landraces from Turkey and Afghanistan, respectively (Fig. 2b). The landraces from Iran formed a diffused group and a few Iranian landraces overlapped with the other 3 groups. We also computed pairwise average F_ST_ (population differentiation coefficient^35^) between different subpopulations. Overall, the F_ST_ analysis indicated a moderate genetic differentiation between MV and LR (Fst = 0.101). The pairwise Fst values, as shown in Fig. 2b, between MV and LR from Turkey, Afghanistan and Iran were 0.101, 0.090 and 0.101, respectively. The pairwise Fst value between LR from Afghanistan and Iran was 0.141 and between LR from Turkey and Iran was 0.142, while it was slightly higher between LR from Afghanistan and Turkey (Fst = 0.151) (Fig. 2b). The genetic diversity parameter, as estimated by PIC, was 0.31, 0.30 and 0.27 for the complete panel, MV and LR, respectively. The nucleotide diversity parameter π was 0.39, 0.38 and 0.36 respectively, for the complete panel, MV and LR, respectively.

LD decayed to half of its maximum at 1.58 Mb in the complete panel, while it decayed at ~ 1.96 and 1.99 Mb in LR and MV (Fig. S3a–c), respectively. The LD decay curve was also drawn at cut off *r*^*2*^ = 0.1 to allow an easy comparison with various previous studies in wheat (discussed in the Discussion section below). The LD decay at cut off *r*^*2*^ = 0.1 was observed at ~ 5.94 Mb in the complete panel, whereas it was observed at ~ 7.58 and 8.08 Mb in LR and MV (Fig. S3d–f), respectively.

### Marker trait associations (MTA) identified using SNP- and haplotype-based GWAS

We used two approaches for GWAS, SNP- and haplotype-based GWAS, to identify MTA of GY and yield parameters in both environments separately. Below, we have first described MTA identified in the drought season using both GWAS approaches followed by MTA identified in the favorable season. All MTA identified by both GWAS are summarized in Tables 1 and 2 separately. The common genomic regions identified by both GWAS are also described, especially when these were co-located with the known metaQTL for GY or known genes in wheat governing yield-related traits (Table S5).

**Table 1:** Marker trait associations identified by SNP-GWAS in drought affected (2018) and favorable (2019) seasons for grain yield (GY), spike length (SL), spike number (SN), number of spikelets per spike (NSS), harvest index (HI) and thousand grain weight (TGW).

Trait	Marker	Chr	Physical position (bp)	*P* value/R^2^ (%)		Co-localization with selection of signature (*P* value in EigenGWAS), MetaQTL for GY, known genes
				Drought affected season (2018)	Favorable season (2019)	
GY	BS00107250_51	1D	27,689,216*		3.71E−07/4.8	MQTL1D.1
	BS00010946_51	1D	488,976,932		3.30E−07/4.9	
	wsnp_Ex_c38739_46195930	2B	192,364,804		9.43E−07/4.5	
	Excalibur_s113043_59	3A	61,308,322*	5.07E−05/10.6		1.05E−16, MQTL3A.2
	wsnp_Ex_rep_c66685_65003254	3A	571,407,719	2.65E−05/11.0		*TaERF3-3A* (Jia et al. 2021)
	BS00000445_51^b^	3A	625,533,783		1.25E−07/5.2	
	BS00001478_51^c^	3A	627,254,114		3.29E−07/4.8	
	AX-94479553	3A	700,811,389		4.98E−07/4.7	
	AX-158598372^b^	3B	562,443,713	1.95E−05/11.1		*TaERF3-3B* (Jia et al. 2021)
	Ku_c101932_436^b^	3B	562,444,883	2.03E−05/11.1		*TaERF3-3B* (Jia et al. 2021)
	wsnp_Ex_c36937_44788679^c^	3B	698,617,994**		4.21E−06/4.7	MQTL26
	wsnp_Ex_c7451_12757458	3D	611,252,106	6.03E−05/10.4		
	Excalibur_c42667_427	3D	611,918,428	4.93E−05/10.2		
	BS00041735_51	4A	594,283,620		1.65E−06/4.4	
	AX-158524359^b^	4A	603,380,455**	2.63E−05/10.9		8.33E−25, MQTL31
	AX-158524348^b^	4A	603,450,276**	2.81E−06/11.1		MQTL31
	RAC875_c59673_188	4A	681,669,144*		1.23E−06/4.3	MQTL4A.3
	BS00067150_51	5A	609,243,940	4.38E−05/10.3		
	wsnp_Ex_c20440_29511162	5B	462,143,612	9.77E−06/11.8		
	BS00063175_51	6A	479,890,385		1.30E−06/4.5	*TaAGL6-A* (Kong et al. 2021)
	AX-89582418	6B	439,070,773	5.99E−05/10.2		2.24E−12
	AX-95136614	6B	702,209,887		1.88E−07/4.4	
	AX-158592462^c^	7B	730,227,625**	1.05E−05/11.6		MQTL63, *TaSBEIb* (Schönhofen et al. 2017)
SL	AX-95245523^c^	1B	15,745,730		1.24E−06/10.9	
	AX-95182696^b^	1B	15,748,142		5.93E−07/13.7	
	AX-110042022^b^	1B	574,277,209*	4.80E−06/10.8		MQTL1B.3
	AX-95254907^c^	1B	576,219,498*	8.08E−06/10.4		MQTL1B.3
	AX-158523658	3A	653,510,868	1.11E−05/10.1		
SL	wsnp_Ex_c14202_22145805	3A	659,159,798	1.14E−05/10.1		1.64E−09
	IACX5899	3A	659,529,512	1.14E−05/10.1		1.64E−09
	AX-94796364	5A	439,878,001***		6.54E−06/11.0	MQTL5A.4, *DEP1-5A* (Li et al. 2022)
	AX-94664659	5B	13,361,005		6.96E−06/11.0	
	Tdurum_contig49841_618^b^	5B	38,166,722*		3.99E−08/15.6	MQTL5B.1
	AX-158525835	5B	559,771,793	6.14E−06/10.6		
	AX-95114986	7B	340,653,213		8.64E−06/9.2	
	wsnp_Ex_c32905_41484291^b^	7B	732,651,100**		2.64E−07/13.7	MQTL63
	BS00042111_51^d^	7B	733,596,201**	1.73E−05/10.4	2.49E−07/13.4	MQTL63
	GENE−4848_559^b,^^c^	7B	739,931,176		1.25E−11/19.6	
SN	IAAV5505^c^	3B	242,747,403	8.52E−05/7.4*		MQTL32
	Kukri_c57965_109	5A	537,127,759**	6.46E−05/6.7		MQTL41
	BS00064947_51	5A	631,268,120	3.21E−05/7.0		
	Tdurum_contig81548_426	5B	632,154,961**	9.21E−05/6.2		MQTL45
	AX-158539210	5D	480,184,657**	1.66E−05/8.8		2.61E−10, MQTL47
	RFL_Contig2815_1305	6A	797,823		8.41E−06/3.1	
	wsnp_Ex_c8741_14630167^b^	6A	522,618,612		1.64E−06/3.5	
	TA006111-0352	6B	713,511,907		3.05E−06/3.4	
	AX-158543927	7B	568,649,768	6.85E−05/7.8		2.73E−13
	BS00023023_51^b^	7B	683,445,856*	8.96E−05/6.4		MQTL7B.5
	RAC875_c13942_2973	7D	93,498,826		2.69E−06/3.4	
NSS	Kukri_c21008_657^b^	2A	779,881,857*	9.34E−05/11.8		MQTL2A.3
	RAC875_c25271_138^a^	2B	762,518,995		7.30E−05/10.7	*TaARF12-2B* (Li et al. 2022)
	Excalibur_c48404_59^b^	2B	789,868,993**		3.17E−05/11.7	MQTL18
	wsnp_Ex_c15646_23969140^b^	2B	789,869,145**		2.92E−05/11.7	MQTL18
	BS00081578_51	2D	67,552,797***		8.69E−05/12.3	*FRIZZY PANICLE−2D*, MQTL2D.5
	AX-158548368	3B	738,748,940	2.34E−05/14.0		
	TG0127	5A	586,725,629		9.13E−05/10.5	
	AX-111624408	5B	700,917,443	4.97E−05/10.3		
	Tdurum_contig43566_801	6A	594,988,644**	3.80E−06/15.7		MQTL49
HI	GENE−0235_131	1A	381,316,740	3.91E−05/12.1		*TaSnRK23-1A* (Miao et al. 2017)
	AX-158556633	1D	493,638,930	1.61E−05/13.0		
	AX-158575330	2B	17,631,084	4.72E−05/12.2		
	RAC875_c25271_138^a^	2B	762,518,995	3.30E−10/24.0		*TaARF12-2B* (Li et al. 2022)
	AX-158523686	3A	649,544,403		2.23E−05/13.1	
	AX-94492274	3B	279,702,676	8.17E−05/11.2		
	AX-89551965^b^	3B	557,097,177		5.57E−05/12.9	
	AX-94504714	5A	669,584,446	9.33E−05/9.1		
	BS00074429_51^c^	6A	2,221,127		4.85E−07/18.0	
	AX-95230651	6D	67,510,483		4.55E−05/12.3	
	RFL_Contig2531_987	7A	10,193,624***		6.44E−05/11.9	*TaGS3-7A*, MQTL7A.1
	AX-110462419	7A	262,463,706	1.94E−05/10.9		
	AX-108837168	7A	674,607,818*		9.93E−06/14.1	MQTL7A.7, *WAPO1* (Kuzay et al. 2019)
	AX-94439426	7A	674,801,909*		2.98E−05/12.7	MQTL7A.7, *WAPO1* (Kuzay et al. 2019)
	RAC875_rep_c78007_394^c^	7B	701,339,824*	8.76E−05/11.3		MQTL7B.2
	Excalibur_c13094_523	7D	235,982,962*	8.34E−05/9.3		MQTL7D.1, *TaSPL16-7D* (Cao et al. 2019)
TGW	Tdurum_contig29983_490	2A	259,213	1.26E−06/9.2		
	AX-95116218	2A	759,732,578*		2.12E−05/6.1	MQTL2A.3, *TaARF12-2A* (Li et al. 2022)
	AX-108882320	3A	720,435,586	9.56E−06/9.1		*TaTGW6-A1* (Hanif et al. 2016)
	Kukri_c23743_112	5B	622,629,693		1.70E−05/6.0	
	Tdurum_contig5017_993^b^	5B	635,358,608**	8.24E−07/9.6		MQTL45
	wsnp_Ex_c99215_85409445	6A	72,432,063*		6.33E−05/5.9	MQTL6A.1
	AX-158552200	6A	447,631,757	2.90E−06/8.7		
	Excalibur_c15844_1470	6A	447,833,875	51.7E−06/11.5		
	AX-94971944	6A	448,230,743	1.91E−06/8.9		
	AX-158552203	6A	449,640,723	1.91E−06/8.9		
	AX-158526868	6A	452,959,034	6.06E−07/9.9		*TaTPP-6A* (Zhang et al. 2017)

*Chr* Chromosome.

^a^Pleiotropic SNPs showing association with multiple traits under the same or different seasons.

^b^SNPs falling in haplotype blocks associated with traits; representation of common genomic regions between the two GWAS.

^c^SNPs identified within 2 Mb of the associated haplotype blocks.

*Meta-QTL of Liu et al. (2020).

**Meta-QTL of Acuña-Galindo et al. (2015).

***Meta-QTL of Saini et al. (2022).

**Table 2:** Marker trait associations identified by haplotype-GWAS in drought affected (2018) and favorable (2019) seasons for grain yield (GY), spike length (SL), spike number (SN), number of spikelets per spike (NSS), harvest index (HI) and thousand grain weight (TGW).

Trait	Haplotype block*	Chr	Physical position (bp)—first to last SNP in the block	*P* value/R^2^ (%)		Co-localization with selection of signature (*P* value in EigenGWAS) or MetaQTL for GY or known genes
				Drought affected season (2018)	Favorable season (2019)	
GY	H1A-42	1A	38,729,484–39,407,691	5.23E−04/16.7		
	H1A-43	1A	45,581,400–46,238,712		1.23E−05/7.7	
	H1B-26	1B	108,837,056–109,729,572		3.63E−05/6.7	
	H1D-26	1D	49,422,527–50,296,844		1.41E−05/6.8	9.61E−30
	H1D-30	1D	272,541,461–272,541,472	5.57E−04/11.3		
	H2A-71	2A	507,068,286–508,033,171***	7.46E−04/16.3		1.22E−18, MQTL2A.2
	H2A-84	2A	686,857,812–686,877,681		4.58E−06/8.0	7.66E−14
	H2A-87	2A	693,292,831–693,421,350	2.20E−04/13.3		
	H2A-97	2A	709,833,346–709,836,593	1.97E−05/17.8		
	H2B-138	2B	683,028,883–683,047,437*	6.67E−04/17.3		MQTL2B.5
	H2B-198	2B	785,229,422–786,105,977*		2.58E−05/7.4	MQTL2B.5
	H2D-22	2D	78,083,628–79,055,437*		2.80E−05/7.4	MQTL2D.4
	H2D-34	2D	579,201,885–580,194,720		3.19E−05/6.8	
	H3A-17^a^	3A	23,642,562–23,829,202*		1.48E−06/7.0	MQTL3A.1
	H3A-90^b^	3A	624,946,738–625,533,783		7.48E−07/8.1	
	H3B-112^b^	3B	562,443,713–562,444,883	5.88E−05/11.4		*TaERF3-3B* (Jia et al. 2021)
	H3B-157^c^	3B	698,607,594–699,509,692**		1.45E−06/7.6	3.55E−15, MQTL26
	H4A-48^b^	4A	603,286,138–603,380,455**	4.75E−04/17.7		8.33E−25, MQTL 31
	H4A-49^b^	4A	603,450,276–603,460,389**	1.33E−05/11.4		MQTL 31
	H4A-63^a^	4A	622,200,839–622,237,185		1.19E−07/7.4	
	H5B-69	5A	456,500,530–457,342,644*		1.84E−05/6.7	MQTL5A.5
	H5B-95	5A	531,538,511–531,591,516		9.65E−07/8.5	4.61E−40
	H5B-171	5A	617,820,739–617,829,157		5.30E−07/6.7	
	H5B-173	5A	620,018,405–620,827,450		4.45E−06/6.5	
	H5D-15	5D	549,852,162–550,151,965*		2.27E−06/8.2	MQTL5D.1
	H6A-47	6A	61,887,622–61,888,224*	4.72E−04/10.4		MQTL6A.1
	H6A-163	6A	614,164,320–614,586,125		1.84E−05/6.7	
	H6B-66	6B	206,831,865–207,388,128***		2.29E−07/7.9	MQTL6B.10, *TaSPL21-6B* (Zhang et al. 2017)
	H6D-8	6D	454,655,598–454,934,087***		6.69E−07/6.6	1.65E−16, MQTL6D.1
	H6D-18	6D	461,316,633–461,413,027***		6.89E−05/7.0	MQTL6D.1
	H7B-24	7B	59,179,319–59,642,066		1.32E−05/6.8	
	H7B-60	7B	552,780,328–553,628,525	3.87E−04/14.5		
	H7B-123	7B	724,940,015–725,380,411**	5.51E−04/12.3		8.83E−29, MQTL63
	H7B-124	7B	727,634,048–727,634,326**	1.70E−04/13.6		MQTL63, *TaSBEIb* (Schönhofen et al. 2017)
	H7B-127^c^	7B	730,151,518–730,152,759**	1.92E−04/17.1		MQTL63, *TaSBEIb* (Schönhofen et al. 2017)
	H7B-128^c^	7B	730,154,257–730,179,871**	6.22E−05/20.1		MQTL63, *TaSBEIb* (Schönhofen et al. 2017)
SL	H1B-7^b^	1B	15,745,280–15,748,142		8.62E−06/21.6	
	H1B-113^b^	1B	573,567,500–574,277,209*	3.40E−05/15.8		6.24E−16, MQTL1B.3
	H3A-146	3A	742,470,065–742,470,253		5.41E−07/17.5	3.38E−14
	H3B-69^a^	3B	241,273,847–242,168,693	3.66E−04/17.1		
	H3B-154	3B	691,454,854–691,750,221**	1.34E−04/16.0		1.71E−20, MQTL26
	H5A-60	5A	462,154,966–463,066,915*		2.68E−07/24.8	9.21E−23, MQTL5A.5
	H5B-3	5B	8,343,384–8,945,985		1.89E−06/23.0	
	H5B-25^b^	5B	37,370,018–38,180,259*		4.54E−06/17.5	4.33E−16, MQTL5B.1
	H5B-113	5B	550,851,238–551,736,975		2.46E−10/30.4	
	H6D-25	6D	464,739,680–465,207,141***	8.93E−04/16.1		MQTL6D.1
	H7A-45^a^	7A	54,943,867–55,345,966	1.69E−04/16.5		7.95E−16
	H7B-109	7B	704,114,527–704,270,130	1.44E−04/15.2		
	H7B-131^b,^^c^	7B	732,651,100–732,653,814**		3.21E−06/16.0	MQTL63
	H7B-132^b^	7B	739,931,176–739,931,859		2.97E−10/20.2	
	H7B-135^c^	7B	741,572,238–741,573,528		7.95E−09/18.9	
SN	H2B-144	2B	692,465,836–692,468,875*		3.92E−07/5.4	MQTL2B.5, *KAT-2B* (Chen et al. 2020)
	H2D-16	2D	73,571,362–74,279,445		3.99E−06/5.3	
	H3A-17^a^	3A	23,642,562–23,829,202*		1.02E−06/5.7	MQTL3A.1
	H3A-102	3A	683,239,168–683,249,841		7.47E−06/3.8	
	H3B-69^a^^,^^c^	3B	241,273,847–242,168,693	8.26E−04/14.6		
	H4A-63^a^	4A	622,200,839–622,237,185	9.42E−04/10.6	1.83E−06/5.3	
	H4A-73	4A	666,148,980–666,151,081*		6.65E−06/3.9	MQTL4A.2
	H6A-78^b^	6A	522,618,612–522,618,750		4.88E−06/4.2	
	H6A-123	6A	599,911,928–600,476,173**		5.49E−07/7.0	8.71E−27, MQTL49, *TaOGT1* (Fan et al. 2021)
	H7A-45^a^	7A	54,943,867–55,345,966	4.34E−04/14.0		7.95E−16
	H7B-36	7B	155,727,255–156,707,640		2.89E−07/6.3	
	H7B-96^b^	7B	683,445,840–683,514,740*	6.99E−04/11.6		2.67E−12, MQTL7B.5
NSS	H1B-3	1B	4,347,096–5,158,560	6.22E−08/32.8		
	H1B-123	1B	614,184,398–614,791,382	2.34E−07/33.3		
	H2A-26	2A	59,553,585–59,554,468		8.02E−04/9.5	*trs1/WFZP-A* (Du et al. 2021)
	H2A-152^a^	2A	761,306,730–761,307,264*		5.55E−04/12.3	3.0E−19, MQTL2A.3, *TaARF12-2A* (Li et al. 2022)
	H2A-161^a^^,b^	2A	779,881,836–780,715,720*	3.10E−09/34.9		6.02E−13, MQTL2A.3
	H2B-2	2B	1,331,398–2,109,745	3.13E−07/31.0		
	H2B-80	2B	192,364,438–192,364,804		9.37E−04/8.0	
	H2B-202^b^	2B	789,868,993–789,869,145**		1.14E−04/11.7	MQTL18
	H3A-8	3A	11,893,292–12,253,793	2.56E−06/30.5		
	H3B-50	3B	69,604,748–70,585,887		4.58E−04/12.5	
	H3B-191	3B	775,824,676–776,359,464**	8.68E−08/31.4		9.65E−15, MQTL27
	H3B-192	3B	778,277,449–778,297,183**	8.07E−09/28.1		MQTL27
	H3B-193^a^	3B	779,135,885–779,535,750**	2.51E−07/33.2		MQTL27
	H3B-194	3B	779,536,788–779,577,828**	3.02E−08/29.2		MQTL27
	H3B-195	3B	781,043,145–781,044,112**	6.70E−10/27.9		MQTL27
	H3B-196	3B	781,044,508–781,493,870**	1.17E−07/28.8		MQTL27
	H4B-57	4B	538,270,213–538,999,562		6.83E−04/13.1	
	H5A-106	5A	535,733,671–536,677,294	1.83E−08/29.7		
	H6A-35	6A	29,610,252–30,190,396	4.37E−07/32.6		
	H6D-15	6D	460,465,066–460,570,638***	7.91E−08/33.5		MQTL6D.1
	H7A-23	7A	19,899,388–19,899,713		1.21E−04/14.1	
	H7A-92	7A	141,261,164–141,272,130		4.89E−04/11.3	*TaPIN1-7A* (Yao et al. 2021)
HI	H1A-19	1A	12,505,528–12,506,155	3.93E−05/18.6		
	H2A-152^a^	2A	761,306,730–761,307,264*	8.15E−09/25.5		3.0E−19, MQTL2A.3, *TaARF12-2A*
	H2A-161^a^	2A	779,881,836–780,715,720*	9.14E−04/18.5		6.02E−13, MQTL2A.3
	H2D-26	2D	81,299,053–81,305,597		2.64E−05/14.5	
	H2D-58	2D	650,322,702–650,325,224		9.85E−06/20.9	
	H3B-10	3B	6,754,335–6,754,382		5.55E−05/15.1	
	H3B-108^b^	3B	557,088,909–557,097,177		1.93E−05/20.1	
	H3B-114	3B	564,760,298–565,637,495	4.72E−04/15.4		
	H3B-126	3B	581,266,296–581,703,874		7.87E−05/24.6	
	H5A-83	5A	492,618,192–493,606,424		2.34E−05/23.2	3.33E−13
	H5A-110	5A	547,814,036–548,609,945	9.08E−04/21.3		2.07E−21
	H5B-68	5B	455,735,459–455,738,860		8.78E−05/25.4	
	H5B-196	5B	678,452,476–678,529,123	4.30E−07/31.1		
	H5B-200	5B	680,357,525–680,605,514		6.94E−05/24.7	
	H6A-5^c^	6A	2,953,239–3,206,299	3.53E−06/25.7		
	H6B-65	6B	201,205,772–201,222,999		9.83E−05/23.3	3.26E−11
	H6B-90	6B	470,807,503–471,167,088		5.24E−05/25.1	
	H7A-186	7A	721,223,720–721,659,295		9.14E−05/25.3	
	H7B-103^c^	7B	701,302,817–701,326,473*	1.48E−04/16.9		MQTL7B.2
	H7D-11	7D	101,398,126–101,761,180	1.73E−05/22.8		
TGW	H1A-145	1A	544,170,158–544,365,169		2.01E−04/10.8	1.06E−12
TGW	H1B-175	1B	680,862,984–680,867,496	5.22E−05/12.9		
	H2A-67	2A	501,850,118–502,623,346***	3.29E−05/11.0		MQTL2A.2, *TaCwi-A1-2A* (Ma et al. 2012)
	H3A-93	3A	651,627,162–651,627,389	6.40E−05/11.3		2.66E−15
	H3A-105	3A	685,357,867–686,127,701		3.40E−06/11.9	
	H3B-147	3B	672,867,140–673,850,338		2.99E−05/10.7	
	H3B-166	3B	723,791,754–724,750,829	7.31E−06/13.8		
	H3B-193^a^	3B	779,135,885–779,535,750**	5.76E−05/16.8		MQTL27
	H5B-161	5B	601,336,331–602,244,923**		9.93E−05/11.3	MQTL44
	H5B-175^b^	5B	635,358,608–635,358,677**	7.85E−06/11.2		MQTL45
	H6A-22	6A	17,911,219–18,713,269	4.58E−05/17.0		

*Chr* chromosome.

^a^Pleiotropic SNPs showing association with multiple traits under the same or different seasons.

^b^Representation of common genomic regions between the two GWAS.

^c^Haplotype blocks identified within 2 Mb of the associated SNPs.

*Meta-QTL of Liu et al. (2020).

**Meta-QTL of Acuña-Galindo et al. (2015).

***Meta-QTL of Saini et al. (2022).

**Figure 3:**
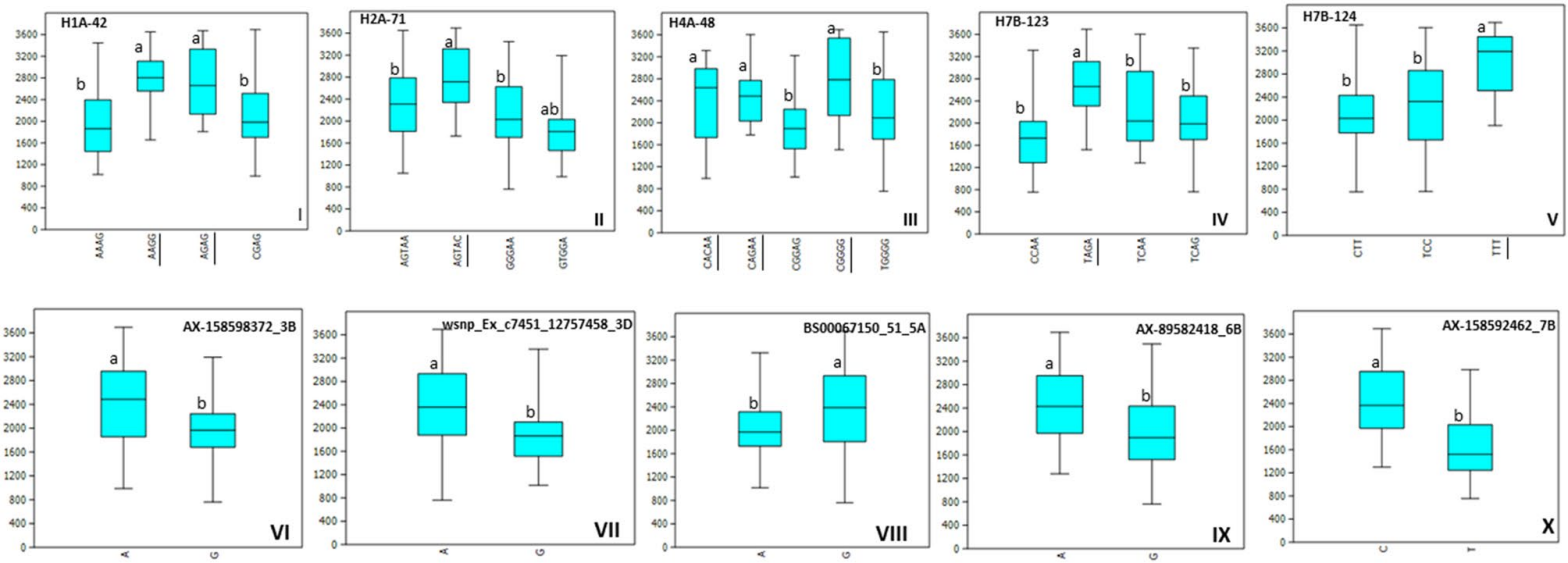
Allelic effects of five best haplotypes (H1A-42, H2A-71, H4A-48, H7B-123 and H7B-124) and five best SNPs (AX-158598372_3B, wsnp_Ex_c7451_12757458_3D, BS00067150_51_5A, AX-89582418_6B and AX-158592462_7B) associated with GY in the drought season. The favorable haplotype alleles are underscored.

**Figure 4:**
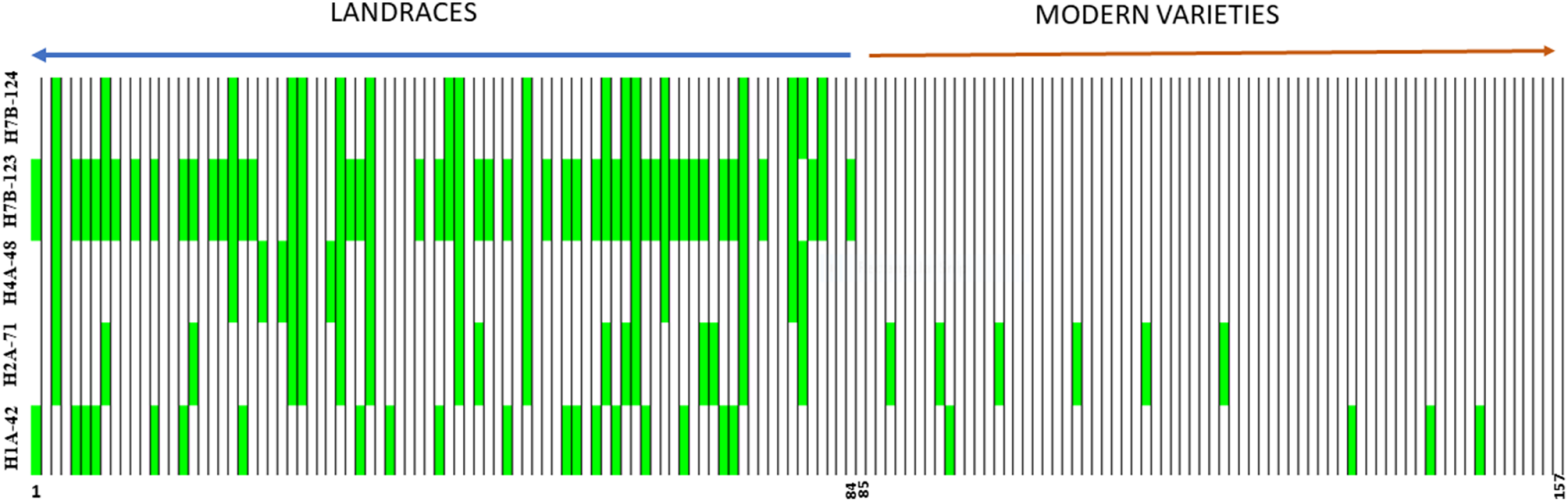
Heat map of the panel showing distribution of favorable haplotype alleles as green vertical rectangle for GY in LR and MV at five best haplotype blocks (H1A-42, H2A-71, H4A-48, H7B-123 and H7B-124). Each vertical black line represents an individual accession and each green vertical rectangle represents the favorable allele of the corresponding haplotype block labelled on the left. The numbers on the x-axis show first and last serial number of the LR (1–84) and MV (85–157) corresponding to the serial number in Supplementary Table S1.

A total of 47 MTA were identified for all traits using SNP-based GWAS in the drought season of 2018. For GY, 12 MTA were identified in SNP-GWAS with percentage variation (PV) varying from 10.2 to 11.8% (Table 1). Haplotypes-GWAS, on the other hand, identified 15 MTA associated with GY with PV ranging from 10.4 to 20.1% (Table 2). Interestingly, a hot spot region (724,940,015–730179871 Mb) was identified on chromosome 7B by haplotype-GWAS, where four haplotypes (H7B-123, H7B-124, H7B-127 and H7B-128) were associated with GY (Table 2). Figure 3 shows the estimated yield advantage at the five best haplotype blocks and the five best SNPs. The favorable alleles at these five haplotype blocks showed invariably yield increase of > 700 kg/ha (Fig. 3I–V), whereas the favorable alleles at the best five SNPs (Fig. 3VI–X) showed yield advantage varying from 352 kg/ha with SNP BS00067150_51_5A) to 710 kg/ha with SNP AX-158592462_7B. Figure 4 shows heat map of the panel showing the distribution of favorable alleles at the five best haplotype blocks in LR and MV. Only ten MV showed a favorable allele at two of the five best haplotypes. Clearly, MV are devoid of favorable alleles of the best five haplotypes identified here. Table S6 shows 16 important landraces that have been identified to carry favorable alleles of two or more than two haplotypes for GY. These 16 landraces showed an average GY of 3436 kg/ha in the drought season and are important for introgression of high allele effect haplotypes into modern varieties. Both GWAS approaches identified common genomic regions for GY on chromosomes 3B, 4A and 7B, of which the genomic regions identified on chromosomes 4A and 7B were located within the two metaQTL regions reported for GY (MQTLs 31 and 63) (Table S5). The genomic region identified on chromosomes 3B was located in the *TaERF3-3B* gene, which plays an important role in grain size and development (Table S5).

For the three spike measurements SL, SN and NSS, SNP-GWAS identified 7, 7 and 4 MTA while haplotype-GWAS identified 6, 7 and 14 MTA, respectively (Tables 1, 2). Most notably for NSS, a 5.6 Mb genomic region (775,824,676–781,493,870 Mb) was identified on chromosome 3B by haplotype-GWAS, where 6 haplotype blocks (H3B-191—H3B-196) were associated with NSS with very high PV varying from 27.9 to 33.2%. The common genomic regions identified by both GWAS for the three spike traits were on chromosomes 1B (SL), 3B and 7B (SN), and 2A (NSS) (Table S5) and these were located within three metaQTL regions (MQTLs 1B.3, 3B.5, 7B.5 and 2A.3) reported for GY under drought or heat stress environments (Table S5). For HI, nine MTA each were identified by both GWAS with PV varying from 9.1 to 24.0% in SNP-GWAS and 16.9 to 31.1% in haplotype-GWAS. A common genomic region was identified on chromosomes 7B (Table S5), which was located in metaQTL7B.2 reported for GY. Interestingly, on chromosome 6A, a constitutive MTA (identified in both seasons) was detected for HI by SNP- and haplotype-GWAS (Table S5), which could not be mapped to any known metaQTL or gene and hence an interesting candidate for future studies. Eight and seven MTA were identified for TGW by SNP- and haplotypes-GWAS with PV varying from 8.7 to 11.5% and 11.0 to 17.0%, respectively. A common genomic region on chromosome 5B, identified by both approaches for TGW, was located in metaQTL45 for GY. Most importantly, three known TGW genes, *TaTGW6-A1* (chromosome 3A), *TaTPP-6A* (chromosome 6A) and *TaCwi-A1-2* (chromosome 2A), were identified to be associated with TGW in the drought season by a combination of both GWAS (Tables 1, 2).

In the favorable season of 2019, 11 and 21 MTA were identified for GY with SNP- and haplotype-GWAS, respectively (Tables 1, 2). Two common regions were identified on chromosomes 3A and 3B by both GWAS (Table S5), of which the genomic region identified on chromosome 3B fell within metaQTL26 for GY. In addition, two important genes governing yield-related traits were identified for GY on chromosomes 6A (*TaAGL6-A*) and 6B (*TaSPL21-6B*) by SNP- and haplotype-GWAS, respectively (Tables 1, 2). For the three spike measurement traits, 9, 4 and 5 MTA and 9, 9 and 8 MTA were identified by SNP- and haplotype-GWAS for SL, SN and NSS, respectively. The common genomic regions identified for the three spike traits by both GWAS were on chromosomes 1B, 5B and 7B for SL, 6A for SN and 2B for NSS (Table S5). Of these, the genomic regions on chromosomes 5B and 7B for SL were in metaQTL5B.1 and metaQTL63, respectively, and the genomic region on chromosome 2B for NSS was in metaQTL18. For HI, SNP- and haplotype-GWAS identified 7 and 11 MTA, respectively, with a common genomic region on chromosomes 3B (557,088,909–557,097,177 Mb), which could not be mapped to any known metaQTL for GY or gene. Three and four MTA were identified for TGW by SNP- and haplotype-GWAS, respectively, and no common genomic region was detected by two GWAS for TGW. Two MTA by SNP-GWAS and one MTA by haplotype-GWAS could be mapped within known metaQTL (Tables 1, 2).

### Comparison of SNP and haplotype-based GWAS

**Figure 5:**
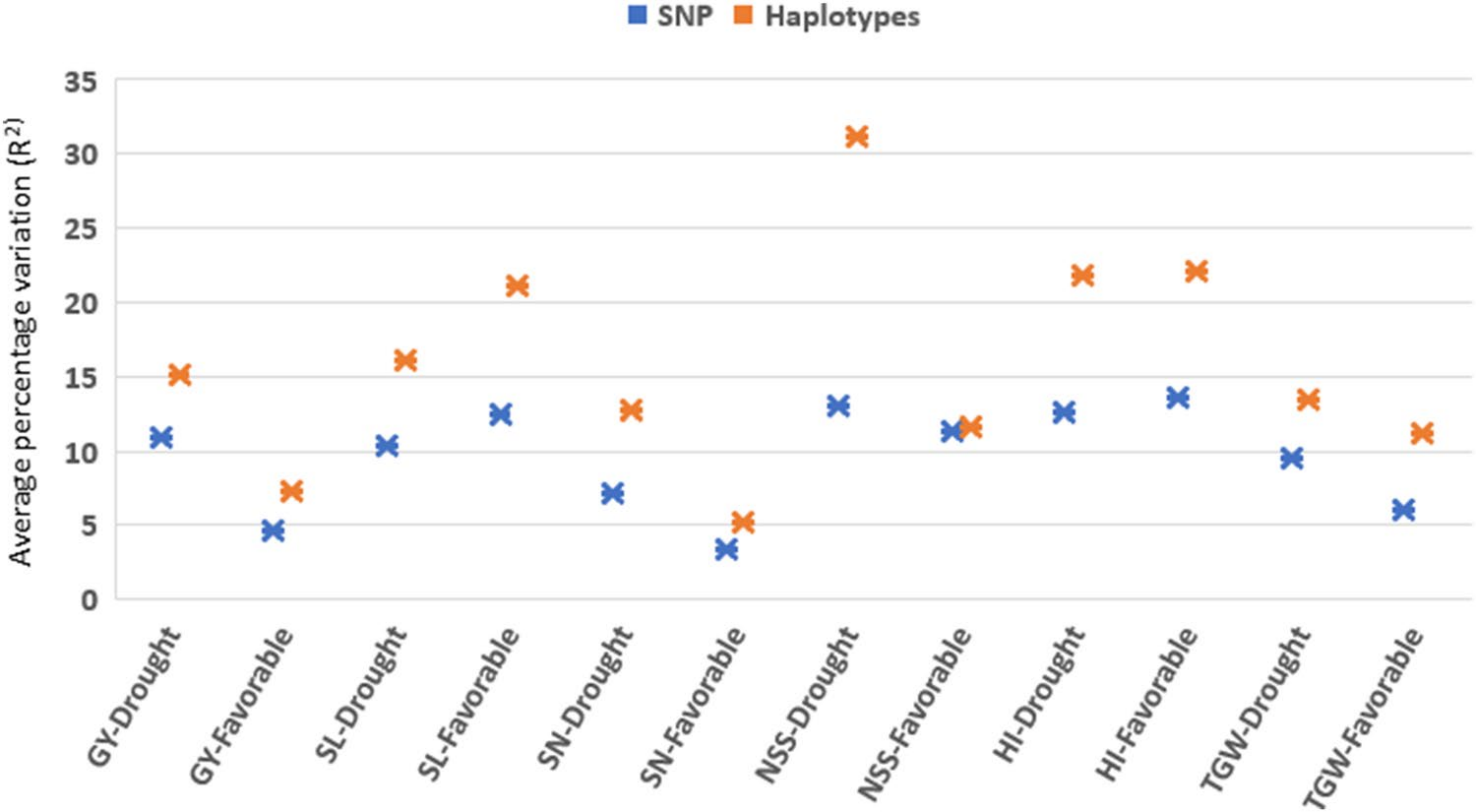
Percentage variation explained by SNPs and haplotypes associated with the traits. *GY* grain yield, *SL* spike length, *SN* spike number, *NSS* number of spikelet per spike, *HI* harvest index, *TGW* thousand grain weight.

A comparison of both approaches showed that the haplotype-GWAS was more effective in identifying MTA with high PV values as compared to SNP-GWAS. For example, haplotype based GWAS identified 15 MTA for GY in the drought season, of which 7 haplotypes showed high PV values of more than 15.0% and 8 haplotypes showed moderate PV values between 10.0 and 14.5%. The SNP-based GWAS, on the other hand, identified 12 MTA in the drought season and all 12 MTA showed moderate PV values of 10.2 to 11.8%. The average PV explained for GY by all associated haplotypes was 15.0 and 7.3%, whereas it was 10.8 and 4.6% by SNPs in the drought and favorable seasons, respectively. A similar trend i.e., higher PV explained by haplotype blocks as compared to SNPs was observed for all the traits in both seasons except NSS in favorable season (Fig. 5).

### Signatures of selection by EigenGWAS and Fst analyses in LR and MV

**Figure 6:**
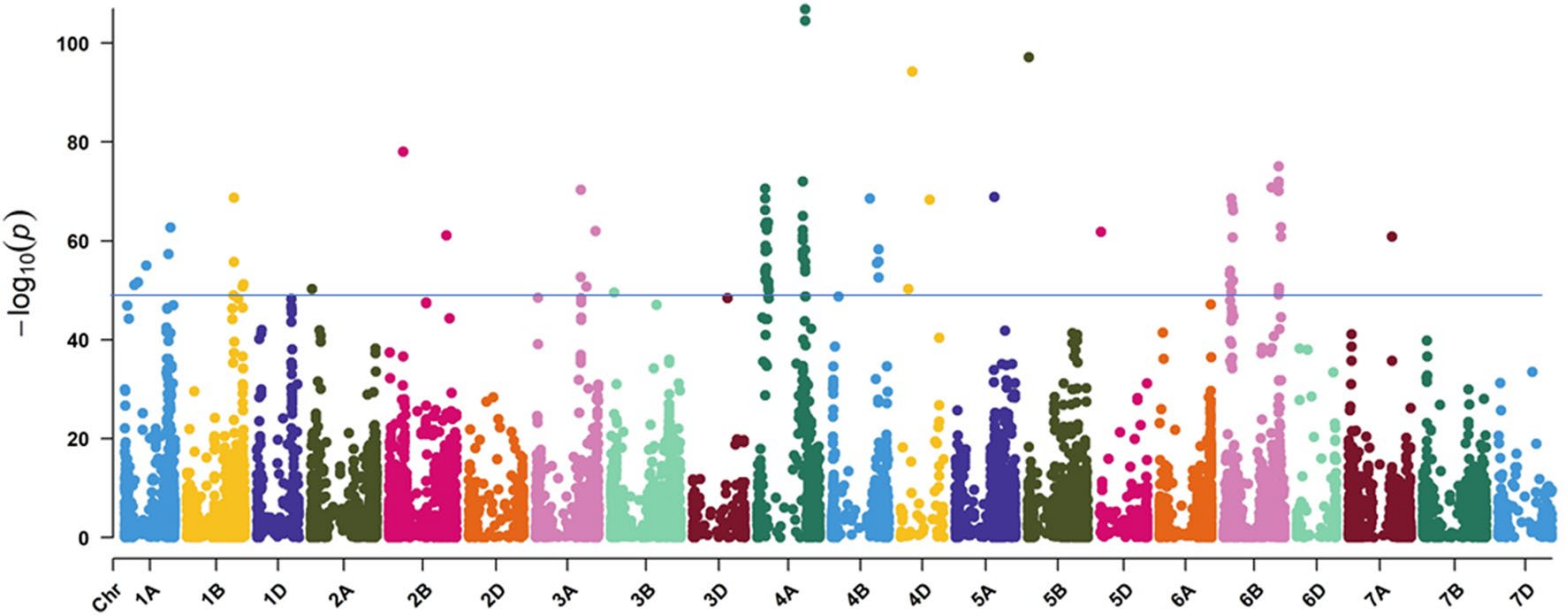
Manhattan plot from EigenGWAS in a panel of Turkish landraces and modern varieties highlighting SNPs that showed signatures of selection. The X-axis represents chromosome numbers and Y-axis represents the corrected P value, also called PGC.

**Figure 7:**
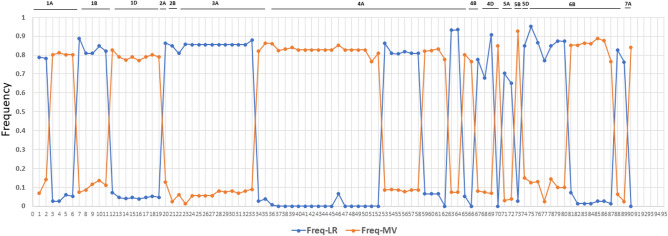
Frequencies of alleles in LR and MV at 90 loci showing signature of selection.

We conducted EigenGWAS and F-statistical test (*F*_ST_) to identify genomic regions that have been differentially selected between LR and MV, hence must have played an important role in adaptation or other selected traits. Overall, EigenGWAS identified 90 SNPs with significant PGC (PGC < 0.01) values (Table S7, Fig. 6). At all loci, one of the SNP alleles was almost fixed in either LR or MV (Fig. 7). The pattern of contrasting allele frequencies in the LR and MV at each of the 90 loci supports the fact that these loci are under differential selection (Fig. 7, Table S7).

In the F-statistical test, SNPs above the threshold of *F*_st_ > 0.101 were declared to be under differential selection based on the average *F*_st_ 0.101 ± 0.007 (SD) between LR and MV. A total of 85 SNPs showed *F*_st_ > 0.101 and 82 of these were common with EigenGWAS (Table S7). The A genome showed the largest number of SNPs under selection (54) followed by the B (25) and D (11) genomes. Based on the genome wide LD threshold of ~ 6 Mb, SNPs within 6 Mb region were merged to declare a selection region and thus 39 selection regions were identified. Notably, on chromosomes 4A and 6B, multiple selection hot spot regions were observed (Fig. 6, Table S7). On chromosomes 4A, five selection regions were identified. Of these, four selection regions were 0.04 Mb (100,634,209–100,673,242 bp), 6.0 Mb (113,854,870–119,931,428), 2.8 Mb (542,827,852–545,618,766) and 0.02 Mb (570,265,418–570,478,671) long, while the fifth region was at 640 Mb identified by one SNP (AX-89422359). Likewise, on chromosome 6B, seven selection regions were evident; three regions were identified by only one SNP at each region at 77.7, 112.9 and 567.4 Mb while the remaining four were 4.2 Mb (92,433,077–96,648,725), 5.4 Mb (103,667,878–109,143,478), 6.5 Mb (650,045,929–656,565,987) and 0.01 Mb (678,175,501–678,344,340) long.

We aligned known genes, published QTL/meta-QTL and MTA identified in GWAS studies in wheat for flowering-time, adaptation and yield and yield-related traits to understand the biological relevance of the selection regions identified here. Table 3 shows selection regions linked to known genes/meta-QTL known for plant adaptation and yield related traits. Out of 39 selection regions, 15 (i.e., 38.4%) were within the proximity of known functional genes controlling flowering [*PRR-A1*, *PPR-D1*, *TaHd1-6B*) and yield and yield components (*TaSus2*-2B, *TaGS2-B1*, *AG1-1A/WAG1-1A*, *DUO-A1*, *DUO-B1, AG2-3A/WAG2-3A*, *TaLAX1, TaSnRK210-4A, FBP, TaLAX1*, *TaPIL1* and *AP3-1-7A/WPA3-7A*). Six selection regions were close (within 5 Mb) to QTL/metaQTL reported for yield and yield related traits. Eight selection regions showed homology with genes coding for ion/amino acid/sugar transporters, transcription factors and regulatory genes (Table S7). For the remaining selection regions, annotated candidate genes were identified from the EnsemblPlant database, however, their molecular functions are not known.

**Table 3:** Known genes/QTL, meta-QTL for plant adaptation and yield traits identified by EigenGWAS and Fst analysis in the present study.

SNP	Chr	Physical position	Allele in LR	Allele in MV	P	PGC	Freq LR	Freq MV	Strenth of selection	Fst	Co-location with known genes/QTL/MetaQTL for yield	Traes ID
Kukri_c44738_477	1A	49,363,611	T	G	6.18E−45	0.0031	0.78	0.10	0.828	0.113	QTL for thousand kernel weight and kernel traits (Bhatta et al. 2018, Li et al. 2019)	TraesCS1A02G067900
AX-158555559	1A	159,173,047	A	C	2.36E−52	0.0014	0.03	0.81	1.260	0.126	proximity to AG1-1A/WAG1-1A gene	
Ra_c21676_178	1A	506,282,302	G	A	4.23E−47	0.0025	0.05	0.80	1.118	0.111	proximity to DUO-A1 gene	
Excalibur_c23155_327	1A	506,553,332	C	T	5.48E−47	0.0025	0.05	0.80	1.136	0.126	proximity to DUO-A1 gene	
Kukri_c24570_282	1A	539,964,551	G	T	2.11E−63	0.0004	0.89	0.07	1.333	0.133	proximity to TaSS4-1A	
AX-110606361	1B	558,910,249	G	A	1.9E−69	0.0002	0.81	0.09	1.061	0.126	proximity to DUO-B1 gene	
AX-95152246	1B	559,575,971	G	A	2.01E−56	0.0009	0.81	0.09	0.965	0.097	proximity to DUO-B1 gene	
IAAV2018	2B	168,622,486	G	A	9.22E−79	7.96E−05	0.85	0.02	1.389	0.139	proximity to TaSus2-2B gene	
Excalibur_c2484_2113	2B	717,474,767	C	T	4.82E−45	0.0031	0.00	0.75	1.192	0.129	proximity to TaGS2-B1 gene	
Tdurum_contig33100_127	3A	535,219,515	T	C	4.62E−71	0.0002	0.86	0.01	1.469	0.147	proximity to AG2-3A/WAG2-3A gene	TraesCS3A02G301800
BS00022882_51	3A	535,224,341	C	T	2.02E−53	0.0012	0.86	0.06	1.295	0.129	proximity to AG2-3A/WAG2-3A gene	TraesCS3A02G301800
BobWhite_c30232_154	3A	535,323,709	A	G	2.02E−53	0.0012	0.86	0.06	1.295	0.129	proximity to AG2-3A/WAG2-3A gene	TraesCS3A02G302100
wsnp_BE443568A_Ta_2_1	3A	536,637,519	T	C	2.02E−53	0.0012	0.86	0.06	1.295	0.129	proximity to AG2-3A/WAG2-3A gene	TraesCS3A02G302700
AX-108817109	3A	537,744,597	T	C	2.02E−53	0.0012	0.86	0.06	1.295	0.129	proximity to AG2-3A/WAG2-3A gene	TraesCS3A02G303400
Tdurum_contig83663_371	3A	540,662,354	G	A	1.11E−44	0.0032	0.86	0.08	1.210	0.121	proximity to AG2-3A/WAG2-3A gene	TraesCS3A02G304600
Kukri_c68006_282	3A	540,665,020	G	A	3.40E−45	0.0031	0.86	0.08	1.227	0.123	proximity to AG2-3A/WAG2-3A gene	TraesCS3A02G304600
Kukri_c47643_920	3A	540,666,242	A	G	1.11E−44	0.0032	0.86	0.08	1.210	0.121	proximity to AG2-3A/WAG2-3A gene	TraesCS3A02G304600
BS00021871_51	3A	540,669,147	T	G	4.17E−49	0.002	0.86	0.07	1.252	0.125	proximity to AG2-3A/WAG2-3A gene	TraesCS3A02G304600
wsnp_BE490613A_Ta_2_1	3A	540,969,715	G	A	1.11E−44	0.0032	0.86	0.08	1.210	0.121	proximity to AG2-3A/WAG2-3A gene	TraesCS3A02G304900
GENE−3939_653	3A	541,209,947	G	A	3.04E−48	0.0022	0.88	0.09	1.247	0.125	proximity to AG2-3A/WAG2-3A gene	TraesCS3A02G305100
wsnp_Ku_c44716_51926415	3A	600,008,239	G	A	1.89E−51	0.0015	0.03	0.82	1.283	0.128	proximity to TaLAX1 gene	
wsnp_CAP8_c296_283066	4A	100,634,209	G	A	2.6E−71	0.0002	0.01	0.86	1.470	0.147	FBP gene	TraesCS4A02G093100
IAAV3906	4A	113,854,870	T	C	1.93E−64	0.0004	0.00	0.83	1.397	0.140	proximity to PRR-A1 flowering gene	
wsnp_Ku_c16481_25377573	4A	114,487,263	T	C	1.93E−64	0.0004	0.00	0.83	1.397	0.140	proximity to PRR-A1 flowering gene	
wsnp_Ku_c7197_12439299	4A	114,587,218	T	C	1.93E−64	0.0004	0.00	0.83	1.397	0.140	proximity to PRR-A1 flowering gene	
wsnp_Ex_c4286_7734046	4A	114,744,423	C	T	1.93E−64	0.0004	0.00	0.83	1.397	0.140	proximity to PRR-A1 flowering gene	
wsnp_Ex_c1387_2659020	4A	115,912,802	A	G	5.84E−63	0.0004	0.00	0.83	1.388	0.139	proximity to PRR-A1 flowering gene	
wsnp_Ku_c14803_23225628	4A	115,913,316	T	C	1.93E−64	0.0004	0.00	0.83	1.397	0.140	proximity to PRR-A1 flowering gene	
wsnp_Ku_c50991_56423564	4A	116,473,035	T	G	8.42E−53	0.0013	0.07	0.85	1.239	0.124	proximity to PRR-A1 flowering gene	
wsnp_Ex_c3178_5868813	4A	116,473,523	C	T	1.93E−64	0.0004	0.00	0.83	1.397	0.140	proximity to PRR-A1 flowering gene	
wsnp_Ex_c27088_36309449	4A	119,071,282	A	C	1.93E−64	0.0004	0.00	0.83	1.397	0.140	proximity to PRR-A1 flowering gene	
wsnp_Ex_c8131_13753986	4A	119,084,973	G	T	1.93E−64	0.0004	0.00	0.83	1.397	0.140	proximity to PRR-A1 flowering gene	
Kukri_c57687_182	4A	119,931,428	C	A	1.93E−64	0.0004	0.00	0.83	1.397	0.140	proximity to PRR-A1 flowering gene	
Kukri_c48155_158	4A	120,605,054	T	G	7.38E−59	0.0007	0.00	0.81	1.343	0.134	proximity to PRR-A1 flowering gene	
AX-108900808	4A	542,827,852	G	C	9.62E−73	0.0002	0.86	0.09	1.208	0.121	TaSnRK210-4A	
AX-158524430	4A	544,389,263	G	C	6.92E−61	0.0005	0.81	0.09	1.057	0.106	TaSnRK210-4A	
TA001512-0387	4A	545,601,781	A	G	9.15E−62	0.0005	0.81	0.09	1.055	0.106	TaSnRK210-4A	
Ra_c37920_342	4A	545,602,051	T	C	9.99E−66	0.0003	0.82	0.08	1.121	0.112	TaSnRK210-4A	
BobWhite_rep_c65013_174	4A	545,603,625	C	T	6.74E−63	0.0004	0.81	0.09	1.061	0.106	TaSnRK210-4A	
AX-158581338	4A	545,618,766	G	A	6.74E−63	0.0004	0.81	0.09	1.061	0.106	TaSnRK210-4A	
Kukri_c48199_102	4B	78,021,175	A	G	1.86E−49	0.0019	0.00	0.77	1.225	0.122	TaSnRK210-4B	
RAC875_c13639_2159	4D	139,205,215	C	T	5.94E−95	1.41E−05	0.91	0.07	1.412	0.141	TaNHX1-4D	
wsnp_Ra_c13906_21872355	4D	341,924,750	G	T	5.03E−69	0.0002	0.00	0.85	1.454	0.145	proximity to TaPRR-4D	
AX-94466267	5A	457,881,918	C	A	1.37E−69	0.0002	0.70	0.03	0.983	0.098	MQTL 57 of Liu et al. 2020 (drought and heat stress)	
AX-158565171	5A	580,799,490	T	C	1.38E−42	0.0041	0.65	0.04	0.847	0.085	proximity to TaPIL1	
Excalibur_c74858_243	5B	13,190,688	A	G	7.53E−98	1.04E−05	0.03	0.93	1.618	0.162	QTL for awn length (Bhatta et al. 2018)	TraesCS5B02G013300
Kukri_c34173_518	5B	531,540,179	C	T	4.17E−42	0.0043	0.85	0.09	0.976	0.121	QTL for spike length (Li et al. 2019)	TraesCS5B02G350900
AX-94999151	5D	13,717,517	C	T	1.57E−62	0.0005	0.95	0.08	1.346	0.135	Proximity to Pina-D1 (3.5 Mb) and Pinb-D1 (3.6 Mb)	TraesCS5D02G020800
AX-95120637	6B	567,470,504	T	C	1.62E−71	0.0002	0.01	0.85	1.422	0.142	TaHd1-6B	
AX-158535361	6B	650,045,929	A	G	1.01E−72	0.0002	0.01	0.86	1.460	0.146	MQTL67 of Liu et al. 2020 (Drought and Heat Stress)	TraesCS6B02G375600
GENE−4566_348	6B	651,411,545	T	C	8.47E−71	0.0002	0.01	0.86	1.454	0.145	MQTL67 of Liu et al. 2020 (Drought and Heat Stress)	TraesCS6B02G376400
Kukri_c3292_670	6B	651,418,760	A	G	9.77E−76	0.0001	0.03	0.89	1.492	0.149	MQTL67 of Liu et al. 2020 (Drought and Heat Stress)	TraesCS6B02G376500
RFL_Contig1105_1309	6B	651,419,105	G	A	2.84E−72	0.0002	0.03	0.88	1.451	0.145	MQTL67 of Liu et al. 2020 (Drought and Heat Stress)	TraesCS6B02G376500
AX-94465863	6B	656,565,987	T	A	7.43E−43	0.004	0.01	0.77	1.177	0.126	MQTL67 of Liu et al. 2020 (Drought and Heat Stress)	TraesCS6B02G381900
wsnp_Ex_c42836_49314564	7A	515,006,480	G	A	1.33E−61	0.0005	0.00	0.84	1.434	0.143	Proximity to AP3-1-7A/WPA3-7A	

*Chr* chromosome, *Freq LR* frequency of allele in landraces, *Freq MV* frequency of allele in modern varieties, *PGC p* value with a genomic control based on EigenGWAS, *Traes ID* ID for annotated genes in wheat in EnsemblPlant database.

Most importantly, the selection regions also included known genes for quality (*Pina-D1*, *Glu-D1*, *TaSS4-1A* and *Tamyb10-A1*) and disease resistance (*Yr78*, *Yr5*) (Table S7). We validated some of the selection regions by genotyping the panel with 32 gene-based KASP assays available for known genes related to flowering, yield related traits, quality and disease resistance in wheat and calculated the allele frequencies and Fst in both groups (Table S8). The results clearly showed that seven genes out of 32 showed signatures of selection (Fst > 0.101) and 5 (*PRR73-A1-4A*, *TaSus2-2B*, *Glu-D1*, *Pinb-D1* and *Yr5*) of these were also identified in EigenGWAS (Table S8). In addition, *Rht-B1* and *Ppd-D1* genes showed signatures of selection but these were missed in EigenGWAS probably due to low density of SNPs around these two genes in this study. Interestingly, LD analysis of the 7 genes revealed that these were in high LD with each other (Fig. S4). Table S9 shows gene-based association mapping for all traits in the two seasons.

## Discussion

GWAS has been extensively deployed in wheat to detect genomic regions associated with complex agronomic traits^38^, however, the approach has not been explored much in combination with selective sweep analysis^14,39^. While GWAS effectively identifies large-effect loci, its application is limited to the germplasm and phenotypes used in analyses and it often misses the opportunity to detect allelic changes associated with signatures of breeding selection^40^. Insights gained from a joint GWAS and selective sweep analysis expand the opportunities to exploit both associated loci and selection footprints for designing effective breeding strategies^14^. The explosion of SNPs in the post genomics era, the concomitant advancements in statistical tools and the availability of high-resolution reference genomes in wheat have provided unprecedented opportunities to perform such integrated analyses and to efficiently apply the data for genomics-assisted breeding.

In the present study, we have characterized a panel of Turkish winter wheat landraces (LR) and modern varieties (MV) by GWAS and signatures of selection analyses to identify the genes and MTA associated with improved yield under drought for deployment in breeding. The LR collection investigated here is a representative set of superior landraces, drawn from a large collection done by IWWIP almost a decade ago from 2009 to 2014^41^. All traits showed significant variation in the panel, supporting the inclusion of all traits in GWAS and signature of selection analyses. The GY in the drought season of 2018 was lower than reported in previous studies on winter wheat germplasm in Konya, Turkey^12,42^. A comparison of yield-related traits such as TGW and HI also revealed significant reductions in these parameters in the present study (16–23% in TGW and 6.0–7.0% in HI) as compared to previous studies (10–12.0% in TGW and 2.0–3.0% in HI), suggesting a more devastating effect of drought in 2018 than previous years^12,42^. The total rainfall during 2018 growing season (149.6 mm) was 32.7 and 38.4% lower than that during 2016 (222.4 mm) and 2017 (243.0 mm) growing seasons in Konya^12^. A comparison of broad sense heritability (*H*^*2*^) of GY and TGW with previous studies in both spring and winter wheat revealed a similar trend as found here^10–12,42,43^, i.e., a low *H*^*2*^ for GY and high *H*^*2*^ for TGW across years. With regards to HI, the present and previous studies showed variable results and our results were in line with those which showed lower *H*^*2*^ for HI^44^. The correlations of traits with GY were higher and significant under drought conditions in both LR and MV than in favorable environment, as was also observed in a previous study^45^. The highest positive correlation between HI and GY in LR suggested that HI is a primary determinant of GY under stress in landraces, whereas in MV it was the spike length. The panel characterized in this study showed high genetic diversity, with an average PIC of 0.31, comparable to winter wheat sets from Australia, Kazakhstan or Croatia^46–48^ and higher than observed in panels of winter wheat germplasm from the US^49–51^. The PIC and π statistics in MV were comparable to the values estimated in the LR, indicating that the genetic diversity of the MV is maintained during crop improvement processes. The International Exchange germplasm Set contains 50% of breeding lines derived from the IWWIP breeding program through partnership with CIMMYT. The diversity in CIMMYT germplasm has been reported to be high through routine introductions of synthetics and other wheat wild relatives^52,53^. The LD decay observed was at ~ 5.94 Mb for the complete panel at cut off *r*^*2*^ = 0.1, which is in the range reported for highly diverse germplasm sets^18,54–57^. The panel revealed a clear distinction between LR and MV, suggesting LR as valuable genetic resource for introgression of novel alleles into MV. Further, Afghan and Turkish landraces formed two distinct groups whereas Iranian landraces overlapped these two groups. This indicates genetic differentiation of Afghan and Turkish landraces and interchange of Iranian landraces with neighboring countries through seed exchange.

SNP- and haplotype-based GWAS were used to identify genomic regions associated with improved GY and yield components under rainfed conditions. We used an LD-based approach to construct genome wide haplotype blocks. The LD-based approach reflects recombination history of the population and thus is the best among all the methods developed to construct haplotype blocks^28^. The average number of haplotype blocks per chromosome was similar to the findings in a synthetic panel^14^ and higher than obtained in a panel of central European winter wheat germplasm^58^. We found very few common MTA between favorable and drought stress seasons for all the studied traits with both GWAS methods. Similar results have been found in many studies in which QTL under optimal and stress conditions have been compared and it is attributed to different evolutionary trajectories induced by contrasting environmental conditions leading to activation of different sets of genes^11,14^. Together, both GWAS identified 18 known genes to confer yield advantage in wheat under different water regimes. Of these, the allelic variation in seven genes (*TaERF3, TaSnRK23, TaARF12*, *TaDEP1, TaTGW6, TaSPL21, TaCwi-A1*) has been shown to be associated with agronomic traits^22,59–63^. It was demonstrated recently that two genes, *TaARF12* and *TaDEP1,* encoding an auxin response factor and the G-protein γ-subunit, respectively, control both plant height and grain weight pleiotropically and both genes are positively selected in Chinese cultivars over the course of breeding^22^. Further, they showed that *TaARF12* and *TaDEP1* interact epistatically with *Rht-1* locus, suggesting that plant height and yield traits have been selected simultaneously during modern wheat breeding.

Haplotype-based GWAS identified a higher number of MTA (26) that overlapped with known meta-QTL and/or the signatures of selection (27) identified in the present study when compared to SNP-based GWAS (16 and 8, respectively). The MTA overlapping with signatures of selection (i.e. showing significant *P* values in EigenGWAS) in both GWAS can be potential future breeding targets after validation. Present results corroborate previous studies and reinforce that haplotypes-based GWAS identifies QTL with better statistical significance (i.e. better *P*-values and higher R^2^) than SNPs^12,18,19,30^. We obtained 4 to 18% higher PV for the traits in haplotype-GWAS as compared to SNP-GWAS. Out of the five best high effect haplotype blocks associated with GY in the drought season, four showed signatures of selection (H2A-71, H4A-48, H7B-123 and H7B-124) and hence are interesting breeding targets. The haplotype blocks H7B-123 and H7B-124 are in proximity of the *TaSBEIb* gene, which codes for a starch branching enzyme (SBE) 1,4-alpha-glucan branching enzyme involved in starch biosynthesis. Starch deposition occurs synchronously with grain development in wheat and its accumulation is greatly affected under drought and heat stress conditions because of significant reduction in the activities of the key enzymes involved in the conversion of sucrose to starch including SBE^64,65^. The SNPs in haplotype block H2A-71 fell in the region of *TraesCS2A02G295400* (Table S10). The ortholog of *TraesCS2A02G295400* in rice *OsGIF1* encodes a cell-wall invertase required for carbon partitioning during early grain-filling^66,67^. Recently, the pleiotropic role of *GIF1* gene has been suggested regulating the sizes of stems, leaves and grains in rice^67^. The SNPs in haplotype block H4A-48 showed homologies with various transcription factor genes including Zinc finger C2H2-type (TraesCS4A02G310700), which are known as master regulators of abiotic stress responses in plants such as drought^68^. The gene network analysis of this candidate gene showed that it is interacting with five other genes (Fig. S5) involved in diverse pathways and regulating leaf relative water content, stomatal resistance, harvest index, days to heading and chlorophyll content; a suite of drought-adaptive traits. Similarly, the SNPs in H1A-42 show homologies with regulatory/transcription factor genes belonging to AP2/ERF domain superfamily (TraesCS1A02G058400) and transporters such as sugar phosphate transporter (TraesCS1A02G058600, TraesCS1A02G058700) (Fig. S6). The gene network analysis indicated the involvement of these genes in multiple stress pathways including drought and cold tolerance and disease resistance. The heat map showed that the favorable alleles of these five high affect haplotypes are either missing in modern varieties or present in less than 10% frequency. Hence, their deployment is essential to enhance the existing gene pool for novel drought stress tolerance alleles and for further yield improvement of the modern germplasm.

We used EigenGWAS and Fst analysis between LR and MV, to identify the footprints of selection that are linked to adaptation and yield improvement. We identified selection footprints in 39 genomic regions. Of these, 15 selection regions were within proximity of known functional genes in wheat controlling flowering (*PRR-A1*, *PPR-D1*, *TaHd1-6B*) and yield and related component traits (*TaSus2*-2B, *TaGS2-B1*, *AG1-1A/WAG1-1A*, *DUO-A1*, *DUO-B1, AG2-3A/WAG2-3A*, *TaLAX1, TaNHX1-4D, TaSnRK210-4A, FBP, TaPIL1* and *AP3-1-7A/WPA3-7A*) (Table 3). A comparison between the sets of genes identified by GWAS and signatures of selection analysis reveals unambiguously that the two approaches pulled out entirely different sets of genes, thus expanding the repertoire of genes that can be utilized in breeding. Notably, the frequencies of early flowering alleles were high in MV for all flowering genes showing signatures of selection (*PRR-A1*, *PPR-D1*, *TaHd1-6B*), whereas for the yield-linked genes selection of alleles was quite variable between the landraces and modern germplasm.

For deployment in breeding, two strategies can be followed. In the first strategy, the genes that showed higher favorable allele frequency in LR (for example, *TaSus2*-2B, *DUO-B1*, *AG2-3A/WAG2-3A, TaSnRK210-4A, TaNHX1-4D* and *TaPIL1*) can be deployed for increasing the frequency of favorable alleles in MV through targeted crosses and marker-assisted selection. The second strategy could be allele mining of genes with higher frequencies of favorable alleles in the modern germplasm to identify new allelic variations. For example, genes such as *TaGS2-B1*, *TaLAX1* and *DUO-A1* could be mined for additional SNP variation that could not be captured here and their association with drought adaptive traits could be re-explored as has been done in case of *TaGS2-B1* gene^69^.

Many previous studies in wheat have shown selection signatures for *Vrn-1* loci^14,23,70,71^. In our study and an earlier study^72^, signatures of selection were not observed for *Vrn-1*. All three major *Vrn 1* loci (*Vrn-A1*, *Vrn-B1* and *Vrn-D1*) showed a balancing selection for winter habit alleles in both LR and MV. The present results therefore suggest that, unlike in spring wheat, the contribution of *Vrn-1* loci in shaping the evolution of the winter wheat is not a significant one. The flowering time genes *PRR-A1*, *PRR-D1*, *TaHd1-6B* and *Ppd-D1* showed signatures of selection indicating the important roles these genes play in fine tuning the crop growth cycle of modern germplasm to increase their adaptability to wider cultivation zones of the country (Turkey) and elsewhere (Iran and Afghanistan). The high LD between *PRR-A1* and *Ppd-D1* genes and significant association of *PRR-A1* and *Ppd-D1* with GY and yield related traits (R^2^ of 32.9–58.8%) further confirm their importance in providing yield advantage to MV (Table S9).

Intriguingly, we found selection footprints for only two gene(s) on homoeologous chromosomes. One gene was identified on chromosomes 4A at 113–120 Mb (*PRR-A1*) and 4D at 341 Mb (*PRR-D1*), respectively. The other gene was *DUO-1* on chromosomes 1A (*DUO-1-1A*) and 1B (*DUO-1-1B*). Such observations are common and have been reported in previous studies in wheat^73,74^. It has been suggested that directional selection rarely acts on multiple advantageous mutations across homoeologous regions. This happens to prevent fitness loss that might occur due to simultaneous mutations in the three copies of the genes on homoeologous chromosomes^73^^.^ The study by^74^ showed simultaneous selection of one SNP in *LEC2* (LEAFY COTYLEDON2) gene on chromosome 2A and 2B. Several selective sweep regions were identified on chromosome 4A and 6B, in the present study. Several selective sweeps on chromosome 4A were also identified in wheat germplasm from Iran and Pakistan^23^, suggesting high selection pressure on genes from this chromosome in wheat from multiple geographies. The longest selective sweep identified on chromosome 4A was a 6 Mb region in the vicinity of the *PRR-A1* gene^75^. *PRR-A1* gene is a paralog of *Ppd1* gene for photoperiod insensitivity. This is not surprising considering the important role played by this gene in fine tuning the flowering times of wheat, especially during stress conditions. Another important selection region was in the vicinity of *TaSnRK210-4A* gene, coding for a sucrose non-fermenting 1-related protein kinase and regulating grain weight and spike length in wheat. Interestingly, a selection region on chromosome 4A was identified at 100.6 Mb where candidate gene search showed proteins/domains of unknown function (DUF) 3527. Since it was selected in the MV in very high frequencies, we assume that it must be playing an important role in adapting the plants to new environments. Although no definite role could be ascertained for DUF3527, evidences are being generated for other families of DUF proteins. For instance, expression profiling of DUF4228 genes was investigated in *Arabidopsis* exposed to multiple abiotic stresses (osmotic, salt and cold) and results suggested the involvement of DUF4228 genes in the pathways of plant resistance to abiotic stresses^76^.

## Conclusion

The identification of many favorable haplotypes from landraces associated with improved GY under drought stress conditions indicates that the landraces have considerable potential towards enhancing the existing gene pool for drought stress tolerance. Sixteen landraces have been identified carrying multiple haplotypes alleles and showing GY from 3000 to 3781 kg/ha. These landraces should be deployed in breeding to expand the repertoire of drought tolerance alleles in the current germplasm for further yield improvement. Further, the genes identified in signatures of selection analyses should be subjected to allele mining in the modern germplasm to identify additional, yet unexplored, superior alleles.

### Supplementary Information

Supplementary Information.

## Data Availability

The data is available as supplementary files.
