# Supplementary material for: Genomic wide association study and selective sweep analysis identify genes associated with improved yield under drought in Turkish winter wheat germplasm
Source: Sci Rep. 2024 Apr 10;14:8431. doi: 10.1038/s41598-024-57469-1 (PMC11006659; doi:10.1038/s41598-024-57469-1)
Supplement: Supplementary file 1 — Supplementary Information. [file 41598_2024_57469_MOESM1_ESM.pdf]

Genomic wide association study and selective sweep analysis identify genes associated with improved yield under drought in Turkish winter wheat germplasm

Deepmala Sehgal[*,1,2], Rathan ND[3], Fatih Özdemir[4], Mesut Keser[5], Beyhan Akin[6], Abdelfattah A. Dababat[6], Emrah Koc[6], Susanne Dreisigacker[1], Alexey Morgounov*[7]

1. International Maize and Wheat Improvement Center (CIMMYT), Km. 45, Carretera Mex- Veracruz, El Batan, Veracruz, CP 56237, Mexico
2. Syngenta, Jealott's Hill International Research Centre, Bracknell, Berkshire RG42 6EY, UK
3. Corteva Agriscience, Hyderabad, Telangana, India
4. Bahri Dagdas International Agricultural Research Institute, Konya, Turkey
5. International Center for Agricultural Research in Dry Areas (ICARDA), Ankara, Turkey
6. International Maize and Wheat Improvement Center (CIMMYT), Ankara, Turkey
7. Kazakh Agrotechnical University named after S. Seifullin, Nur-Sultan, 010011 Kazakhstan

Corresponding authors

deepmala.sehgal@syngenta.com, alexey.morgounov@gmail.com

Table S1 List of 157 genotypes; 84 landraces accessions (LR) and 73 modern varieties and breeding lines (MV)

S. No.	Entry name	LOCAL NAME	LR/MV	Origin
1	19LR-IN-EX-43	SARDARI BIOTYPE	LR	IRAN
2	19LR-IN-EX-163	CIRPUZ	LR	TUR
3	19LR-IN-EX-64	KALAK ROBAT SANGI	LR	AFG
4	19LR-IN-EX-160	KUNDURU	LR	TUR
5	19LR-IN-EX-26	SORGÜL	LR	TUR
6	19LR-IN-EX-31	HACIALİ	LR	TUR
7	19LR-IN-EX-46	SARDARI BIOTYPE	LR	IRAN
8	19LR-IN-EX-61	OMID BEDON_E_ DASA	LR	AFG
9	19LR-IN-EX-23	AKYARNAZ	LR	TUR
10	19LR-IN-EX-5	AKBUGDAY	LR	TUR
11	19LR-IN-EX-17	KIRMIZI KUNDURU	LR	TUR
12	19LR-IN-EX-154	AKBUGDAY	LR	TUR
13	19LR-IN-EX-49	SARDARI BIOTYPE	LR	IRAN
14	19LR-IN-EX-162	SIVEREK	LR	TUR
15	19LR-IN-EX-7	CALIBASAN	LR	TUR
16	19LR-IN-EX-33	ŞERGUN	LR	TUR
17	19LR-IN-EX-67	DANDAN SHOTUR	LR	AFG
18	19LR-IN-EX-164	KILCIKSIZ BUGDAY	LR	TUR
19	19LR-IN-EX-159	CAKMAK	LR	TUR
20	19LR-IN-EX-22	GODEREDI	LR	TUR
21	19LR-IN-EX-38	KHOSHEH ABLAQ	LR	IRAN
22	19LR-IN-EX-27	HINTA	LR	TUR
23	19LR-IN-EX-13	KUNDURU	LR	TUR
24	19LR-IN-EX-35	SORGÜL	LR	TUR
25	19LR-IN-EX-57	SURKHAK MAHALI	LR	AFG
26	19LR-IN-EX-156	ORMECE	LR	TUR
27	19LR-IN-EX-37	WHITE SPIKE	LR	IRAN

28	19LR-IN-EX-68	SHANAZE	LR	AFG
29	19LR-IN-EX-155	CAM BUGDAYI	LR	TUR
30	19LR-IN-EX-8	ORMECE	LR	TUR
31	19LR-IN-EX-73	NESH SHOTOR	LR	AFG
32	19LR-IN-EX-50	SARDARI BIOTYPE	LR	IRAN
33	19LR-IN-EX-53	SARDARI BIOTYPE	LR	IRAN
34	19LR-IN-EX-24	KIRMIZI BUĞDAY	LR	TUR
35	19LR-IN-EX-59	SAFEDAK KALAK BEDON_E_ DASA	LR	AFG
36	19LR-IN-EX-9	AGBUGDAY	LR	TUR
37	19LR-IN-EX-19	AKBUGDAY	LR	TUR
38	19LR-IN-EX-29	SORİK	LR	TUR
39	19LR-IN-EX-2	CAM BUGDAYI (KIRMIZI)	LR	TUR
40	19LR-IN-EX-165	KOCA BUGDAY	LR	TUR
41	19LR-IN-EX-74	KALAK	LR	AFG
42	19LR-IN-EX-32	HACIALİ	LR	TUR
43	19LR-IN-EX-70	ROSHAN SAFED KHOSHA	LR	AFG
44	19LR-IN-EX-69	DANDAN	LR	AFG
45	19LR-IN-EX-153	UVEYIK BUGDAYI	LR	TUR
46	19LR-IN-EX-65	ZARDCHA KALAK ROBAT SANGI	LR	AFG
47	19LR-IN-EX-3	AGSUNTERI	LR	TUR
48	19LR-IN-EX-6	ORMECE	LR	TUR
49	19LR-IN-EX-51	SARDARI BIOTYPE	LR	IRAN
50	19LR-IN-EX-21	CIRPUZ	LR	TUR
51	19LR-IN-EX-36	KHOSHEH QERMEZ	LR	IRAN
52	19LR-IN-EX-25	KIRMIZI BUĞDAY	LR	TUR
53	19LR-IN-EX-45	SARDARI BIOTYPE	LR	IRAN
54	19LR-IN-EX-11	CAKMAK	LR	TUR
55	19LR-IN-EX-48	SARDARI BIOTYPE	LR	IRAN
56	19LR-IN-EX-52	SARDARI BIOTYPE	LR	IRAN
57	19LR-IN-EX-15	CIRPUZ	LR	TUR

58	19LR-IN-EX-44	SARDARI BIOTYPE	LR	IRAN
59	19LR-IN-EX-71	OMID KALAK ROBAT SANGI	LR	AFG
60	19LR-IN-EX-34	KIRMIZI BUĞDAY	LR	TUR
61	19LR-IN-EX-66	LALMI BOR KHOSHA	LR	AFG
62	19LR-IN-EX-58	KALAK BOR KHOSHA	LR	AFG
63	19LR-IN-EX-28	ZINEBE	LR	TUR
64	19LR-IN-EX-42	SARDARI BIOTYPE	LR	IRAN
65	19LR-IN-EX-62	OMID SORKH KHOSHA BEDON_E_ DASAH	LR	AFG
66	19LR-IN-EX-10	KUNDURU	LR	TUR
67	19LR-IN-EX-55	SARDARI BIOTYPE	LR	IRAN
68	19LR-IN-EX-4	CAM BUGDAYI (BEYAZ)	LR	TUR
69	19LR-IN-EX-41	SIAHDANEH	LR	IRAN
70	19LR-IN-EX-39	QZIL KHOSHEH	LR	IRAN
71	19LR-IN-EX-54	SARDARI BIOTYPE	LR	IRAN
72	19LR-IN-EX-47	SARDARI BIOTYPE	LR	IRAN
73	19LR-IN-EX-75	KALAK SORKH KHOSHA	LR	AFG
74	19LR-IN-EX-18	CIRPUZ	LR	TUR
75	19LR-IN-EX-14	SIVEREK	LR	TUR
76	19LR-IN-EX-12	CAKMAK	LR	TUR
77	19LR-IN-EX-56	ATTAYE	LR	AFG
78	19LR-IN-EX-76	SAFFRONI	LR	AFG
79	19LR-IN-EX-72	SORKHAK WATANI LALMI	LR	AFG
80	19LR-IN-EX-16	KIRMIZI KUNDURU	LR	TUR
81	19LR-IN-EX-63	LALMI SAFEDAK	LR	AFG
82	19LR-IN-EX-30	HACIALİ	LR	TUR
83	19LR-IN-EX-161	CIRPUZ	LR	TUR
84	19LR-IN-EX-157	POLATLI	LR	TUR
85	19LR-IN-EX-141	ALMALY//PBW343/PASTOR	MV	IRR
86	19LR-IN-EX-101	AZAR2/78ZHONG291-64	MV	SA
87	19LR-IN-EX-110	KS061860M-3	MV	SA

88	19LR-IN-EX-109	OR1/SANTA FE/3/OVERLEY*3/AMADINA//KS990011~27	MV	SA
89	19LR-IN-EX-127	38IBWSN-97/DESTIN	MV	IRR
90	19LR-IN-EX-125	ESKINA-7/3/NEMURA/CRDN//78014-40	MV	IRR
91	19LR-IN-EX-98	SPARTANKA//PBW343*2/KUKUNA	MV	SA
92	19LR-IN-EX-103	FULLER/OVERLEY//KS980554-12-~9	MV	SA
93	19LR-IN-EX-105	X031102-6-4/OK00611W//KS98W0512-2-~4	MV	SA
94	19LR-IN-EX-80	GEREK	MV	SA
95	19LR-IN-EX-112	KS090120C*-25	MV	SA
96	19LR-IN-EX-133	COPPER/7/ZCL/3/PGFN//CNO67/SN64/4/SERI/5/UA.2837/6/ATTILA/3*BCN	MV	IRR
97	19LR-IN-EX-102	KS010567-4-2/KS980512-11	MV	SA
98	19LR-IN-EX-144	RETEZAT	MV	IRR
99	19LR-IN-EX-81	NACIBEY	MV	SA
100	19LR-IN-EX-118	B1551-WH/KS94U326/3/F10S-1//STOZHER/KARL/4/F10S-1//STOZHER/KARL	MV	IRR
101	19LR-IN-EX-91	SHARK/F4105W2.1//CHAKINSKAYA306	MV	SA
102	19LR-IN-EX-138	KRASNOVODOPADSKAYA-25//PBW343*2/KUKUNA	MV	IRR
103	19LR-IN-EX-119	AGRI/NAC//KAUZ/3/1D13.1/MLT/4/ATAY/GALVEZ87//SHARK-1	MV	IRR
104	19LR-IN-EX-135	CMH84.168/4/TAST/SPRW//ZAR/3/ATAY/GALVEZ87	MV	IRR
105	19LR-IN-EX-116	MT0419/DESTIN//BONITO-36	MV	IRR
106	19LR-IN-EX-136	TAM200/3/F60314.76/MRL//CNO79/4/84.40022/5/AGRI/NAC//KAUZ/3/1D13.1	MV	IRR
107	19LR-IN-EX-85	ND643/2*WAXWING/4/TAM200/KAUZ/3/AGRI/BJY//VEE	MV	SA
108	19LR-IN-EX-147	ART/KS990494-11-~O//KS06O3A~36	MV	IRR
109	19LR-IN-EX-88	BILINMIYEN96.55/7/ZCL/3/PGFN//CNO67/SN64/4/SERI/5/UA.2837/6/ATTILA/3*BCN	MV	SA
110	19LR-IN-EX-96	GRK79//PBW343*2/KUKUNA	MV	SA
111	19LR-IN-EX-87	DE9//INQALAB 91*2/TUKURU/3/308.02.2/WEAVER//362K2.121	MV	SA
112	19LR-IN-EX-108	ARS97135-9/O3A-B4//KS06O3A~49	MV	SA
113	19LR-IN-EX-129	PYN*2/CO725052/3/KAUZ*2/YACO//KAUZ/4/KRIA	MV	IRR
114	19LR-IN-EX-93	VORONA//PRL/VEE#6/3/KAUZ/3/*2/YACO//KAUZ//PANTHEON/BLUEGIL-2	MV	SA
115	19LR-IN-EX-150	TX00D1390/RONL//KS990159-3-~7	MV	IRR
116	19LR-IN-EX-142	INTENSIVNAYA/KUKUNA	MV	IRR
117	19LR-IN-EX-115	KONYA 2002	MV	IRR

118	19LR-IN-EX-130	CROC_1/AE.SQUARROSA(205)//KAUZ/3/LUFER/4/KS97P0630-4-5/CM95560//X920879-C15-1/3/X84W063-9-18/U1324-25-1-4-….	MV	IRR
119	19LR-IN-EX-78	KARAHAN-99	MV	SA
120	19LR-IN-EX-89	CA8055/4/ROMTAST/BON/3/DIBO//SU92/CI13645/5/AGRI/BJY//VEES/6/KS9468/NWT//ARKAN/3/PASTOR/7/YE2453//PPBB68/CHRC	MV	SA
121	19LR-IN-EX-149	FULLER/OVERLEY//KS980554-12-~9	MV	IRR
122	19LR-IN-EX-151	KS061880M-3	MV	IRR
123	19LR-IN-EX-126	BABAX/LR42//BABAX*2/3/VIVITSI/4/AGRI/NAC//ATTILA	MV	IRR
124	19LR-IN-EX-107	CO050337-2/BYRD	MV	SA
125	19LR-IN-EX-99	KARLYGASH*2/TUKURU	MV	SA
126	19LR-IN-EX-124	ORH010083/AHMETAGA	MV	IRR
127	19LR-IN-EX-97	SANZAR-8/TUKURU	MV	SA
128	19LR-IN-EX-114	KATE A-1	MV	IRR
129	19LR-IN-EX-95	TAM200/3/F60314.76/MRL//CNO79/4/84.40022/5/AGRI/NAC//KAUZ/3/1D13.1	MV	SA
130	19LR-IN-EX-134	PANTHEON/BLUEGIL-2/5/AGRI/BJY//VEE/3/BUL6687.12/4/F6038W12.1	MV	IRR
131	19LR-IN-EX-143	PARTENER	MV	IRR
132	19LR-IN-EX-137	INTENSIVNAYA/KKTS	MV	IRR
133	19LR-IN-EX-148	O3A-B8//WBLL 1*2/KUKUN/3/FULLER	MV	IRR
134	19LR-IN-EX-121	HATCHER/KS03HW12-1//NUDAKOTA	MV	IRR
135	19LR-IN-EX-77	MUFITBEY	MV	SA
136	19LR-IN-EX-94	VORONA//MILAN/SHA7/3/MV17/4/ATAY/GALVEZ87//SHARK-1	MV	SA
137	19LR-IN-EX-139	GRK79//INQALAB 91*2/TUKURU	MV	IRR
138	19LR-IN-EX-82	TEMPORALERAM87*2/4/HD2281/TRAP#1/3/KAUZ*2/TRAP//KAUZ/5/STEKLOVIDNAYA24/6/F10S-1//STOZHER/KARL	MV	SA
139	19LR-IN-EX-86	ZANDER-17//SAULESKU#26/PARUS	MV	SA
140	19LR-IN-EX-122	F4141-W-1-1/PASTOR//PYN/BAU/4/VORONA//MILAN/SHA7/3/MV17/5/DORADE-5	MV	IRR
141	19LR-IN-EX-123	TAM200*2/MO88//KAMB1*2/KUKUNA/3/SW89-3218/VORONA	MV	IRR
142	19LR-IN-EX-132	CITARI-9/MV18-2000//STARSHINA	MV	IRR
143	19LR-IN-EX-84	ES14/SITTA//AGRI/NAC/5/TRAP#1/YACO/3/KAUZ*2/TRAP//KAUZ/4/KINACI9127/ID800994.W/FALKE	MV	SA
144	19LR-IN-EX-128	BABAX/LR42//BABAX*2/3/KURUKU/4/TX96V2427	MV	IRR
145	19LR-IN-EX-117	T88/2180//T811//KRISTADORA/3/SHARK/F4105W2.1	MV	IRR
146	19LR-IN-EX-146	MV NEMERE	MV	IRR
147	19LR-IN-EX-111	KS080448*C-102	MV	SA

148	19LR-IN-EX-79	SONMEZ01	MV	SA
149	19LR-IN-EX-113	BEZOSTAYA	MV	IRR
150	19LR-IN-EX-152	T-153	MV	IRR
151	19LR-IN-EX-145	MV SED	MV	IRR
152	19LR-IN-EX-106	OCW00M618S-2B/KS020482TM~3//NUHILLS	MV	SA
153	19LR-IN-EX-83	STAR/BWD//ATAY/GALVEZ87	MV	SA
154	19LR-IN-EX-90	KS00F5-14-7/EUREKA//ZARGANA-4	MV	SA
155	19LR-IN-EX-104	ARS97135-9/O3A-B4//KS06O3A~49	MV	SA
156	19LR-IN-EX-92	SULTAN95/ATILLA//ZARGANA-6	MV	SA
157	19LR-IN-EX-131	T136//T812*2/KARL/3/ZUBKOV/3/AGRI/NAC//ATTILA	MV	IRR

Table S2 Mean monthly temperatures and total monthly rainfalls in the 2018 and 2019 growing seasons

Month	Precipitation (mm)		Mean air temperature (^{o}C)	
	2018	2019	2018	2019
January	34.8	66.6	1.3	0.5
February	3.3	31.6	5.7	4.1
March	20.8	35.0	9.8	6.4
April	14.4	32.0	13.9	6.6
May	22.2	10.2	17.2	17.8
June	38.8	45.6	21.2	20.9
Sum/average	149.5	206.8	11.5	9.4

Table S3 Analysis of variance (ANOVA) and broad sense heritability of traits investigated in the study

Trait	Source	Df	MS	F value	*P* value	H^2		
						Drought (2018)	Irrigated (2019)	Combined
GY	Genotype	156	1604631	5.1322	<2.2e-16	0.93	0.40	0.46
	Environment	1	480968263	1538.3108	<2.2e-16			
	Genotype x Environment	156	1512774	4.8384	<2.2e-16			
	Residuals	313	312660					
SL	Genotype	156	6.4985	16.7306	<2.2e-16	0.84	0.73	0.62
	Environment	1	0.0318	0.0817	0.7752			
	Genotype x Environment	155	1.3986	3.6006	<2.2e-16			
	Residuals	280	0.3884					
SN	Genotype	156	17.721	4.6590	<2.2e-16	0.86	0.39	0.42
	Environment	1	283.656	74.5752	4.692e-16			
	Genotype x Environment	152	5.739	1.5089	0.0016372			
	Residuals	277	3.804					
NSS	Genotype	156	3.835	2.5096	1.222e-11	0.65	0.66	0.65
	Environment	1	243.323	159.2354	<2.2e-16			
	Genotype x Environment	152	2.245	1.4694	0.002981			
	Residuals	277	1.528					
HI	Genotype	156	62.4	2.5752	3.321e-12	0.59	0.76	0.48
	Environment	1	4450.9	183.5484	<2.2e-16			
	Genotype x Environment	152	35.6	1.4685	0.003024			
	Residuals	277	24.2					

TGW	Genotype	156	97.2	8.4666	<2.2e-16	0.72	0.81	0.74
	Environment	1	10280.4	895.6764	<2.2e-16			
	Genotype x Environment	152	20.4	1.7803	1.753e-05			
	Residuals	277	11.5					

Table S4 Distribution and genome wide coverage of SNPs, polymorphic information content (PIC) and number of haplotype blocks per chromosomes

Chromosome	No. of SNPs	PIC	Physical genome covered (bp)-First and last SNP across chromosome	Number of haplotype blocks (HB)	Number of SNPs in an HB (min-max)
1A	972	0.31	1174240-593550661	175	2-10
1B	1026	0.30	1203935-689000691	176	2-12
1D	272	0.31	85165-495126574	51	2-19
2A	1068	0.31	259213-780715720	161	2-11
2B	1263	0.30	80838-801253577	205	2-13
2D	353	0.30	1609331-650881432	59	2-10
3A	877	0.30	432514-749404716	148	2-11
3B	1196	0.31	337188-829286660	209	2-12
3D	185	0.29	773182-614367136	22	2-15
4A	581	0.31	3124914-744515299	87	2-8
4B	480	0.29	537211-673252119	88	2-9
4D	58	0.32	1242201-509666790	7	2-10
5A	1131	0.30	10511-708442531	200	2-15
5B	1191	0.31	19616-713016195	225	2-15
5D	192	0.31	344895-563854980	22	2-16
6A	993	0.32	287373-617689725	172	2-21
6B	971	0.32	164066-720985852	169	2-13
6D	195	0.33	1770966-473370071	32	2-11
7A	1195	0.28	534538-736691678	197	2-13
7B	828	0.30	115751-750605774	140	2-14
7D	181	0.28	378699-634890748	23	2-9

Table S5 Similar* and closely linked** (within 2Mb) genomic regions identified by SNP and haplotypes-GWAS for all traits in the two environments

Trait	Marker/haplotype	Chr	Physical position (SNP/haplotype)	P value/R2 SNPs, P value/R2 haplotypes		MetaQTL/known genes
				2018	2019	
**GY**	BS00000445_51 */H3A-90	3A	625533783/624946738-625533783		1.25E-07/5.2, 7.48E-07/8.1	
	BS00001478_51** /H3A-90	3A	627254114/624946738-625533783		3.29E-07/4.8, 7.48E-07/8.1	
	AX-158598372 */H3B-112	3B	562443713/ 562443713- 562444883	1.95E-05/11.1, 5.88E-05/11.4		*TaERF3-3B*
	Ku_c101932_436 */H3B-112	3B	562444883/ 562443713- 562444883	2.03E-05/11.1, 5.88E-05/11.4		*TaERF3-3B*
	wsnp_Ex_c36937_44788679**/H3B-157	3B	698617994/698607594- 699509692		4.21E-06/4.7, 1.45E-06/7.6	MQTL26 (Acuña-Galindo et al. 2015)
	AX-158524359 */ H4A-48	4A	603380455/603286138- 603380455	2.63E-05/10.9, 4.75E-04/17.7		MQTL31 (Acuña-Galindo et al. 2015)
	AX-158524348 */H4A-49	4A	603450276/603450276- 603460389	2.81E-06/11.1, 1.33E-05/11.4		MQTL31 (Acuña-Galindo et al. 2015)
	AX-158592462 **/ H7B-127, H7B-128	7B	730227625/730151518- 730152759, 730154257- 730179871	1.05E-05/11.6, 1.92E-04/17.1, 6.22E-05/20.1		MQTL63 (Acuña-Galindo et al. 2015)
**SL**	AX-95182696 */H1B-7	1B	15748142/15745280- 15748142		5.93E-07/13.7, 8.62E-06/21.6	
	AX-95245523 **/H1B-7	1B	15745730/15745280- 15748142		1.24E-06/10.9, 8.62E-06/21.6	
	AX-110042022 */H1B-113	1B	574277209/573567500- 574277209	4.80E-06/10.8, 3.40E-05/15.8		MQTL1B.3 (Liu et al. 2020)
	AX-95254907 **/H1B-113	1B	576219498/573567500- 574277209	8.08E-06/10.4, 3.40E-05/15.8		MQTL1B.3 (Liu et al. 2020)
	Tdurum_contig49841_618 **/H5B-25	5B	38166722/37370018- 38180259		3.99E-08/15.6, 4.54E-06/17.5	MQTL5B.1 (Liu et al. 2020)
	wsnp_Ex_c32905_41484291 */H7B-131	7B	732651100/732651100-732653814		2.64E-07/13.7, 3.21E-06/16.0	MQTL63 (Acuña-Galindo et al. 2015)
	GENE-4848_559 *, **/H7B-132, H7B-135	7B	739931176/739931176- 739931859, 741572238- 741573528		1.25E-11/19.6, 2.97E-10/20.2, 7.95E-09/18.9	

**SN**	IAAV5505 **/H3B-69	3B	242747403/241273847- 242168693	8.52E-05/7.4*/8.26E-04/14.6		MQTL3B.5 (Liu et al. 2020)
	wsnp_Ex_c8741_14630167 */H6A-78	6A	522618612/522618612- 522618750		1.64E-06/3.5/4.88E-06/4.2	
	BS00023023_51 */H7B-96	7B	683445856/683445840- 683514740	8.96E-05/6.4, 6.99E-04/11.6		MQTL7B.5 (Liu et al. 2020)
**NSS**	Kukri_c21008_657 */H2A-161	2A	779881857/779881836- 780715720	9.34E-05/11.8, 3.10E-09/34.9		MQTL2A.3 (Liu et al. 2020)
	Excalibur_c48404_59 */H2B-202	2B	789868993/789868993- 789869145		3.17E-05/11.7, 2.92E-05/11.7	MQTL18 (Acuña-Galindo et al. 2015)
	wsnp_Ex_c15646_23969140 */H2B-202	2B	789869145/789868993- 789869145		2.92E-05/11.7, 2.92E-05/11.7	MQTL18 (Acuña-Galindo et al. 2015)
**HI**	AX-89551965 */H3B-108	3B	557097177/557088909- 557097177		5.57E-05/12.9, 1.93E-05/20.1	
	BS00074429_51 **/H6A-5	6A	2221127/2953239- 3206299	3.53E-06/25.7	4.85E-07/18.0	
	RAC875_rep_c78007_394 **/H7B-103	7B	701339824/701302817- 701326473	8.76E-05/11.3, 1.48E-04/16.9		MQTL7B.2 (Liu et al. 2020)
**TGW**	Tdurum_contig5017_993 */H5B-175	5B	635358608/635358608- 635358677	8.24E-07/9.6, 7.85E-06/11.2		MQTL45 (Acuña-Galindo et al. 2015)

Chr; Chromosome

Table S6 Sixteen landraces showing two or two or more than two high effect haplotypes for GY.

The favorable allele at each haplotype block is highlighted in green.

**LR**	**H1A-42**	**H2A-71**	**H4A-48**	**H7B-123**	**H7B-124**	**Origin**	**GY 2018 (kg/ha)**
19LR-IN-EX-64	AGAG	AGTAC	CGGGG	TAGA	TTT	WLR-AFG	3618.5
19LR-IN-EX-26	AAGG	AGTAA	TGGGG	TAGA	TCC	WLR-TUR	3225.5
19LR-IN-EX-61	AGAG	AGTAC	TGGGG	TAGA	TTT	WLR-AFG	3249.5
19LR-IN-EX-23	CGAG	AGTAA	TGGGG	TAGA	CTT	WLR-TUR	3366.5
19LR-IN-EX-49	AAGG	AGTAA	TGGGG	TAGA	CTT	WLR-IRAN	3740
19LR-IN-EX-33	AAGG	AGTAA	TGGGG	TAGA	TCC	WLR-TUR	3390.5
19LR-IN-EX-38	AGAG	AGNAC	CGGGG	TAGA	TTT	WLR-IRAN	3006.5
19LR-IN-EX-68	CGAG	AGTAC	CGGGG	TAGA	TTT	WLR-AFG	3781.5
19LR-IN-EX-50	CGAG	AGTAC	CGGGG	TAGA	TTT	WLR-IRAN	3301.5
19LR-IN-EX-59	AGAG	AGTAC	CGGGG	TAGA	TTT	WLR-AFG	3725.5
19LR-IN-EX-70	AAAG	GGGAA	CAGAA	TAGA	TTT	WLR-AFG	3522.5
19LR-IN-EX-48	AAGG	AGTAA	TGGGG	TAGA	CTT	WLR-IRAN	3160
19LR-IN-EX-58	AGAG	AGTAC	CGGGG	TAGA	TTT	WLR-AFG	3396
19LR-IN-EX-62	AGAG	AGKAC	CGGGG	TAGA	TTT	WLR-AFG	3762.5
19LR-IN-EX-39	MGAG	AGTAC	CRGRR	TMRA	TCY	WLR-IRAN	3406.5
19LR-IN-EX-75	CGAG	AGTAC	CGGGG	TAGA	TTT	WLR-AFG	3328

LR; Landrace

Table S7 Signatures of selection identified by EigenGWAS in the present study.

SNP	Chr	Physical pos	Allelel in WLR	Allele in MV	P	Freq WLR	Freq MV	Strenth of selection	Fst	Co-location with known genes/QTL/MetaQTL for yield	TraesID
AX-94803018	1A	33226939	G	C	1.24E-47	0.79	0.07	1.0732	0.117		TraesCS1A02G051600
Kukri_c44738_477	1A	49363611	T	G	6.18E-45	0.78	0.10	0.8283	0.113	QTL for thousand kernel weight, kernel length and kernel number per spike in Bhatta et al. 2018, Li et al. 2019 and Hanif et al. 2021	TraesCS1A02G067900
BS00087437_51	1A	113860372	T	C	8.27E-52	0.03	0.80	1.23	0.123		TraesCS1A02G112100
AX-158555559	1A	159173047	A	C	2.36E-52	0.03	0.81	1.2604	0.126	proximity to AG1-1A/WAG1-1A gene	
Ra_c21676_178	1A	506282302	G	A	4.23E-47	0.05	0.80	1.1184	0.111	proximity to DUO-A1 gene	
Excalibur_c23155_327	1A	506553332	C	T	5.48E-47	0.05	0.80	1.1359	0.126	proximity to DUO-A1 gene	
Kukri_c24570_282	1A	539964551	G	T	2.11E-63	0.89	0.07	1.3329	0.133	proximity to TaSS4-1A	
AX-110606361	1B	558910249	G	A	1.9E-69	0.81	0.09	1.061	0.126	proximity to DUO-B1 gene	
AX-95152246	1B	559575971	G	A	2.01E-56	0.81	0.09	0.9654	0.097	proximity to DUO-B1 gene	
AX-158570571	1B	662154441	G	A	3.23E-47	0.85	0.05	1.0169	0.112		TraesCS1B02G441100
AX-158545108	1B	662669760	T	C	1.87E-51	0.82	0.06	1.0188	0.112		TraesCS1B02G441900
AX-108797365	1B	668126415	A	G	5.63E-52	0.07	0.83	1.145	0.115	proximity to TaRec8-B1 gene	
RAC875_c54260_127	1D	408152562	C	T	5.66E-49	0.05	0.79	1.1283	0.113	proximity to GluD1 gene	
BobWhite_c10977_834	1D	408204578	C	T	4.06E-47	0.04	0.78	1.1082	0.111	proximity to GluD1 gene	
BS00079095_51	1D	408207085	T	G	5.66E-49	0.05	0.79	1.1283	0.113	proximity to GluD1 gene	
IAAV2840	1D	408535224	T	C	2.42E-44	0.04	0.77	1.1068	0.111	proximity to GluD1 gene	
AX-158540395	1D	409050801	G	A	5.66E-49	0.05	0.79	1.1283	0.113	proximity to GluD1 gene	
Excalibur_c8188_243	1D	410467979	C	T	5.48E-47	0.05	0.80	1.1359	0.114	proximity to GluD1 gene	
BS00058554_51	1D	410792235	C	T	3.68E-46	0.05	0.79	1.1242	0.112	proximity to GluD1 gene	
wsnp_Ex_c101374_86744680	2A	16555329	A	G	5.63E-51	0.86	0.08	1.0847	0.108		TraesCS2A02G040200
IAAV2018	2B	168622486	G	A	9.22E-79	0.85	0.02	1.389	0.139	proximity to TaSus2-2B gene	
Excalibur_rep_c66832_742	2B	682848571	G	T	8.98E-62	0.81	0.06	1.143	0.124	proximity to stripe rust gene Yr5, Yr7, YrSP genes	
Excalibur_c2484_2113	2B	717474767	C	T	4.82E-45	0.00	0.75	1.1924	0.129	proximity to TaGS2-B1 gene	

Tdurum_contig33100_127	3A	535219515	T	C	4.62E-71	0.86	0.01	1.469	0.147	proximity to AG2-3A/WAG2-3A gene	TraesCS3A02G301800
BS00022882_51	3A	535224341	C	T	2.02E-53	0.86	0.06	1.2948	0.129	proximity to AG2-3A/WAG2-3A gene	TraesCS3A02G301800
BobWhite_c30232_154	3A	535323709	A	G	2.02E-53	0.86	0.06	1.2948	0.129	proximity to AG2-3A/WAG2-3A gene	TraesCS3A02G302100
wsnp_BE443568A_Ta_2_1	3A	536637519	T	C	2.02E-53	0.86	0.06	1.2948	0.129	proximity to AG2-3A/WAG2-3A gene	TraesCS3A02G302700
AX-108817109	3A	537744597	T	C	2.02E-53	0.86	0.06	1.2948	0.129	proximity to AG2-3A/WAG2-3A gene	TraesCS3A02G303400
Tdurum_contig83663_371	3A	540662354	G	A	1.11E-44	0.86	0.08	1.2097	0.121	proximity to AG2-3A/WAG2-3A gene	TraesCS3A02G304600
Kukri_c68006_282	3A	540665020	G	A	3.40E-45	0.86	0.08	1.2267	0.123	proximity to AG2-3A/WAG2-3A gene	TraesCS3A02G304600
Kukri_c47643_920	3A	540666242	A	G	1.11E-44	0.86	0.08	1.2097	0.121	proximity to AG2-3A/WAG2-3A gene	TraesCS3A02G304600
BS00021871_51	3A	540669147	T	G	4.17E-49	0.86	0.07	1.2516	0.125	proximity to AG2-3A/WAG2-3A gene	TraesCS3A02G304600
wsnp_BE490613A_Ta_2_1	3A	540969715	G	A	1.11E-44	0.86	0.08	1.2097	0.121	proximity to AG2-3A/WAG2-3A gene	TraesCS3A02G304900
GENE-3939_653	3A	541209947	G	A	3.04E-48	0.88	0.09	1.2465	0.125	proximity to AG2-3A/WAG2-3A gene	TraesCS3A02G305100
wsnp_Ku_c44716_51926415	3A	600008239	G	A	1.89E-51	0.03	0.82	1.2825	0.128	proximity to TaLAX1 gene	
AX-94898331	3A	708234805	C	A	1.03E-62	0.04	0.86	1.3657	0.137	proximity to Tamyb10-A1 gene	
wsnp_CAP8_c296_283066	4A	100634209	G	A	2.6E-71	0.01	0.86	1.4704	0.147	*FBP* gene	TraesCS4A02G093100
IAAV3115	4A	100672014	G	A	5.44E-64	0.00	0.82	1.4051	0.141		TraesCS4A02G093300
wsnp_Ex_c2617_4864441	4A	100672728	C	T	6.44E-67	0.00	0.83	1.4117	0.141		TraesCS4A02G093300
wsnp_Ex_c2617_4864955	4A	100673242	T	C	2.8E-69	0.00	0.84	1.4338	0.143		TraesCS4A02G093300
IAAV3906	4A	113854870	T	C	1.93E-64	0.00	0.83	1.397	0.140	proximity to PRR-A1 flowering gene	
wsnp_Ku_c16481_25377573	4A	114487263	T	C	1.93E-64	0.00	0.83	1.397	0.140	proximity to PRR-A1 flowering gene	
wsnp_Ku_c7197_12439299	4A	114587218	T	C	1.93E-64	0.00	0.83	1.397	0.140	proximity to PRR-A1 flowering gene	
wsnp_Ex_c4286_7734046	4A	114744423	C	T	1.93E-64	0.00	0.83	1.397	0.140	proximity to PRR-A1 flowering gene	
wsnp_Ex_c1387_2659020	4A	115912802	A	G	5.84E-63	0.00	0.83	1.3881	0.139	proximity to PRR-A1 flowering gene	
wsnp_Ku_c14803_23225628	4A	115913316	T	C	1.93E-64	0.00	0.83	1.397	0.140	proximity to PRR-A1 flowering gene	
wsnp_Ku_c50991_56423564	4A	116473035	T	G	8.42E-53	0.07	0.85	1.2386	0.124	proximity to PRR-A1 flowering gene	
wsnp_Ex_c3178_5868813	4A	116473523	C	T	1.93E-64	0.00	0.83	1.397	0.140	proximity to PRR-A1 flowering gene	
wsnp_Ex_c27088_36309449	4A	119071282	A	C	1.93E-64	0.00	0.83	1.397	0.140	proximity to PRR-A1 flowering gene	
wsnp_Ex_c8131_13753986	4A	119084973	G	T	1.93E-64	0.00	0.83	1.397	0.140	proximity to PRR-A1 flowering gene	

Kukri_c57687_182	4A	119931428	C	A	1.93E-64	0.00	0.83	1.397	0.140	proximity to PRR-A1 flowering gene	
Kukri_c48155_158	4A	120605054	T	G	7.38E-59	0.00	0.81	1.3433	0.134	proximity to PRR-A1 flowering gene	
AX-108900808	4A	542827852	G	C	9.62E-73	0.86	0.09	1.2084	0.121	TaSnRK210-4A	
AX-158524430	4A	544389263	G	C	6.92E-61	0.81	0.09	1.0567	0.106	TaSnRK210-4A	
TA001512-0387	4A	545601781	A	G	9.15E-62	0.81	0.09	1.0551	0.106	TaSnRK210-4A	
Ra_c37920_342	4A	545602051	T	C	9.99E-66	0.82	0.08	1.1208	0.112	TaSnRK210-4A	
BobWhite_rep_c65013_174	4A	545603625	C	T	6.74E-63	0.81	0.09	1.061	0.106	TaSnRK210-4A	
AX-158581338	4A	545618766	G	A	6.74E-63	0.81	0.09	1.061	0.106	TaSnRK210-4A	
AX-158581336	4A	570265418	A	G	1.62E-54	0.07	0.82	1.1486	0.115		TraesCS4A02G257400
BS00022174_51	4A	570267527	G	T	4.04E-55	0.07	0.83	1.1614	0.116		TraesCS4A02G257400
AX-158542105	4A	570267837	A	G	6.58E-59	0.07	0.83	1.184	0.118		TraesCS4A02G257400
RAC875_c27704_420	4A	570469609	T	C	1.86E-49	0.00	0.78	1.2577	0.126		TraesCS4A02G257700
wsnp_Ex_rep_c104859_89444355	4A	570477911	G	A	3.47E-105	0.93	0.07	1.4721	0.147		TraesCS4A02G257800
BobWhite_rep_c63429_271	4A	570478671	A	G	1.52E-107	0.93	0.07	1.4787	0.148		TraesCS4A02G257800
AX-89422359	4A	640000252	C	A	5.69E-43	0.05	0.80	1.1401	0.114		TraesCS4A02G366600
Kukri_c48199_102	4B	78021175	A	G	1.86E-49	0.00	0.77	1.2247	0.122	*TaSnRK210-4B*	
AX-110791800	4B	535245142	G	T	3.16E-56	0.78	0.08	0.9953	0.113		TraesCS4B02G264600
AX-95073308	4D	89537979	C	T	5.77E-51	0.68	0.07	0.7839	0.121		TraesCS4D02G110100
RAC875_c13639_2159	4D	139205215	C	T	5.94E-95	0.91	0.07	1.4123	0.141	TaNHX1-4D	
wsnp_Ra_c13906_21872355	4D	341924750	G	T	5.03E-69	0.00	0.85	1.4539	0.145	proximity to TaPRR-4D	
AX-94466267	5A	457881918	C	A	1.37E-69	0.70	0.03	0.9826	0.098	MQTL 57 of Liu et al. 2020 (drought and heat stress)	TraesCS5A02G242900, TraesCS5A02G243000, TraesCS5A02G243100
AX-158565171	5A	580799490	T	C	1.38E-42	0.65	0.04	0.8468	0.085	proximity to *TaPIL1*	
Excalibur_c74858_243	5B	13190688	A	G	7.53E-98	0.03	0.93	1.6175	0.162	QTL for awn length (Bhatta et al. 2018)	TraesCS5B02G013300
Kukri_c34173_518	5B	531540179	C	T	4.17E-42	0.85	0.09	0.976	0.121	QTL for spike length (Li et al. 2019)	TraesCS5B02G350900
AX-94999151	5D	13717517	C	T	1.57E-62	0.95	0.08	1.3457	0.135	Proximity to Pina-D1 (3.5Mb) and Pinb-D1 (3.6Mb)	TraesCS5D02G020800
BS00067590_51	6B	77749687	T	C	7.11E-52	0.87	0.09	1.0857	0.112		TraesCS6B02G099800
AX-158624672	6B	92433077	C	T	3.11E-69	0.77	0.02	1.1706	0.117	Yr78	
RAC875_c23812_187	6B	96648725	T	G	4.50E-47	0.85	0.10	0.9939	0.115		TraesCS6B02G114400
AX-158552704	6B	103667878	A	G	5.83E-68	0.88	0.10	1.207	0.121		TraesCS6B02G116500

BobWhite_rep_c64102_331	6B	109143478	T	C	7.79E-67	0.87	0.10	1.2021	0.120		TraesCS6B02G117600
Tdurum_contig43872_549	6B	112905768	T	C	1.20E-52	0.07	0.85	1.2171	0.121		TraesCS6B02G118100
AX-95120637	6B	567470504	T	C	1.62E-71	0.01	0.85	1.4221	0.142	TaHd1-6B	
AX-158535361	6B	650045929	A	G	1.01E-72	0.01	0.86	1.4604	0.146	MQTL67 of Liu et al. 2020 (Drought and Heat Stress)	TraesCS6B02G375600
GENE-4566_348	6B	651411545	T	C	8.47E-71	0.01	0.86	1.4538	0.145	MQTL67 of Liu et al. 2020 (Drought and Heat Stress)	TraesCS6B02G376400
Kukri_c3292_670	6B	651418760	A	G	9.77E-76	0.03	0.89	1.4915	0.149	MQTL67 of Liu et al. 2020 (Drought and Heat Stress)	TraesCS6B02G376500
RFL_Contig1105_1309	6B	651419105	G	A	2.84E-72	0.03	0.88	1.4514	0.145	MQTL67 of Liu et al. 2020 (Drought and Heat Stress)	TraesCS6B02G376500
AX-94465863	6B	656565987	T	A	7.43E-43	0.01	0.77	1.1766	0.126	MQTL67 of Liu et al. 2020 (Drought and Heat Stress)	TraesCS6B02G381900
tplb0021a17_853	6B	678175501	C	A	1.36E-61	0.83	0.06	1.1885	0.119		TraesCS6B02G401700
AX-158588692	6B	678344340	T	C	1.68E-63	0.76	0.02	1.1536	0.115		TraesCS6B02G401900
wsnp_Ex_c42836_49314564	7A	515006480	G	A	1.33E-61	0.00	0.84	1.4338	0.143	Proximity to AP3-1-7A/WPA3-7A	

The regions that did not overlap or fell in proximity of known genes or QTL, gene annotations are provided.

Table S8 Fst values and frequencies of gene-based marker alleles in landraces (LR) and modern varieties (MV). The numbers in bold highlight Fst values of the genes under selection and the frquencies of the SNP alleles that became fixed

**Gene**	**Fst**	**Freq-LR**	**Freq-MV**
Rht-B1	**0.129**	0.04	**0.77**
Rht-D1	0.000	0.00	0.04
Rht8	0.008	0.80	0.63
Ppd-A1	0.001	0.96	0.89
Ppd-B1	0.000	0.04	0.01
Ppd-D1	**0.116**	0.11	**0.64**
Vrn-A1	0.000	0.93	0.92
Vrn-B1	0.006	0.55	0.74
Vrn-D1	0.000	0.19	0.14
TaELF3-D1	0.006	0.99	0.82
PRR73-A1	**0.120**	0.07	**0.85**
TPP-6A	0.005	1.00	0.85
TEF-7A	0.010	0.01	0.23
TaGS5-A1-3A	0.000	0.61	0.52
TaGS-D1-7D	0.006	0.50	0.34
TaSus1-7B	0.000	0.86	0.89
TaSus-7A	0.000	0.29	0.34
TaSus2-2A	0.000	0.74	0.79
TaSus2-2B	**0.117**	**0.64**	0.03
TaCwi-4A	0.006	0.75	0.56
Dreb1-3B	0.027	0.15	0.52
GW2-6B	0.000	1.00	0.99
Glu-A1	0.015	0.67	0.93
Glu-D1	**0.117**	0.07	**0.68**
PPO-D1	0.007	0.44	0.27
Pinb-D1	**0.133**	0.01	**0.52**

Lr14	0.000	0.29	0.30
Lr34	0.018	0.00	0.27
Lr46	0.000	0.38	0.45
Lr68	0.000	0.21	0.23
Yr57	0.000	0.89	0.85
Yr5	**0.110**	0.29	0.00

Table S9 Marker trait associations for gene-based markers identified in the present study. Genes with percentage variation >20% are shown in bold.

**Trait**	**Season**	***Marker***	**Chr**	**Pos**	**marker_F**	**p**	**marker_Rsq**
BLUP_TGW	Drought season	*Rht-B1*	4B	30861571	42.44059	2.58E-15	**0.36292**
BLUP_TGW	Drought season	*PRR73-A1*	4A	1.19E+08	74.78756	7.70E-15	**0.33419**
BLUP_TGW	Drought season	*TaSus2-2B*	2B	1.71E+08	54.30287	1.05E-11	**0.2645**
BLUP_SN	Drought season	*PRR73-A1*	4A	1.19E+08	39.59593	3.30E-09	**0.20995**
BLUP_TGW	Drought season	*Pinb-D1*	5D	3600000	37.9553	6.86E-09	0.2086
BLUP_TGW	Drought season	*Ppd-D1*	2D	33952488	34.62114	2.49E-08	0.18652
BLUP_SN	Drought season	*TaSus2-2B*	2B	1.71E+08	22.62343	4.56E-06	0.1303
BLUP_GY	Drought season	*Rht-B1*	4B	30861571	10.67785	4.56E-05	0.12248
BLUP_GY	Favorable season	*PRR73-A1*	4A	1.19E+08	219.1315	2.47E-31	**0.58886**
BLUP_SN	Favorable season	*PRR73-A1*	4A	1.19E+08	218.5561	2.78E-31	**0.58822**
BLUP_GY	Favorable season	*Rht-B1*	4B	30861571	85.55517	1.15E-25	**0.52794**
BLUP_SN	Favorable season	*Rht-B1*	4B	30861571	78.91384	2.83E-24	**0.50777**
BLUP_PH	Favorable season	*Rht-B1*	4B	30861571	70.11106	2.45E-22	**0.47821**
BLUP_GY	Favorable season	*TaSus2-2B*	2B	1.71E+08	110.7365	7.05E-20	**0.41672**
BLUP_SN	Favorable season	*TaSus2-2B*	2B	1.71E+08	106.219	2.69E-19	**0.40663**
BLUP_GY	Favorable season	*Pinb-D1*	5D	3600000	82.26437	6.75E-16	**0.35726**
BLUP_SN	Favorable season	*Ppd-D1*	2D	33952488	76.31564	3.68E-15	**0.32992**
BLUP_GY	Favorable season	*Ppd-D1*	2D	33952488	73.60247	9.30E-15	**0.32197**
BLUP_SN	Favorable season	*Pinb-D1*	5D	3600000	69.48083	4.88E-14	**0.31948**
BLUP_TGW	Favorable season	*PRR73-A1*	4A	1.19E+08	34.7683	2.29E-08	0.18517
BLUP_TGW	Favorable season	*Rht-B1*	4B	30861571	19.07103	4.03E-08	0.19955
BLUP_TGW	Favorable season	*TaSus2-2B*	2B	1.71E+08	32.99764	4.74E-08	0.17552
BLUP_GY	Favorable season	*Dreb1-3B*	3B	82053887	29.77149	1.90E-07	0.162
BLUP_SN	Favorable season	*Dreb1-3B*	3B	82053887	27.32698	5.51E-07	0.15071
BLUP_PH	Favorable season	*TaSus2-2B*	2B	1.71E+08	25.52689	1.21E-06	0.1414
BLUP_NSS	Favorable season	*Dreb1-3B*	3B	82053887	22.79405	4.16E-06	0.12893
BLUP_DH	Favorable season	*Vrn-D1*	5D	4.67E+08	20.37739	1.25E-05	0.11619

BLUP_GY	Favorable season	*TEF-7A*	7A	66229630	19.53999	1.86E-05	0.11325
BLUP_NSS	Favorable season	*PRR73-A1*	4A	1.19E+08	19.26888	2.11E-05	0.11185
BLUP_SN	Favorable season	*TEF-7A*	7A	66229630	16.65401	7.20E-05	0.09816

Table S10 Candidate genes identified for haplotype blocks H1A-42, H2A-71 and H4A-48 associated with grain yield

**Trait**	**Haplotype block**	**Markers in haplotype block**	**Chr**	**Pos**	**TraesID**	**Candidate genes in wheat/orthologs in other species**	**Molecular function**
GY	H1A-42	wsnp_BE490041A_Ta_2_1	1A	38729484	TraesCS1A02G058400	AP2/ERF domain superfamily	DNA binding, DNA-binding transcription factor activity
		RAC875_c89908_105	1A	38731816			
		RAC875_c826_2186	1A	39407483	TraesCS1A02G058600, TraesCS1A02G058700	Sugar phosphate transporter domain	
		TA003773-0807	1A	39407691			
	H2A-71	AX-110434028	2A	507068286	TraesCS2A02G295400	*OsGIF1*	Glycosyl hydrolase, Glycosyl hydrolase family 32, C-terminal,
		BS00032606_51	2A	507387260			
		Kukri_c11327_977	2A	507691445			
		AX-158540709	2A	507693777			
		TG0136	2A	508033171			
	H4A-48	AX-158524491	4A	603286138	TraesCS4A02G310400	ACT domain-containing protein ACR1-12	
		AX-109818490	4A	603286465			
		AX-158524347	4A	603334618			
		AX-158533355	4A	603373961	TraesCS4A02G310600	PsbP, C-terminal, Mog1/PsbP	calcium ion binding
		AX-158524359	4A	603380455	TraesCS4A02G310700	Zinc finger C2H2-type	nucleic acid binding

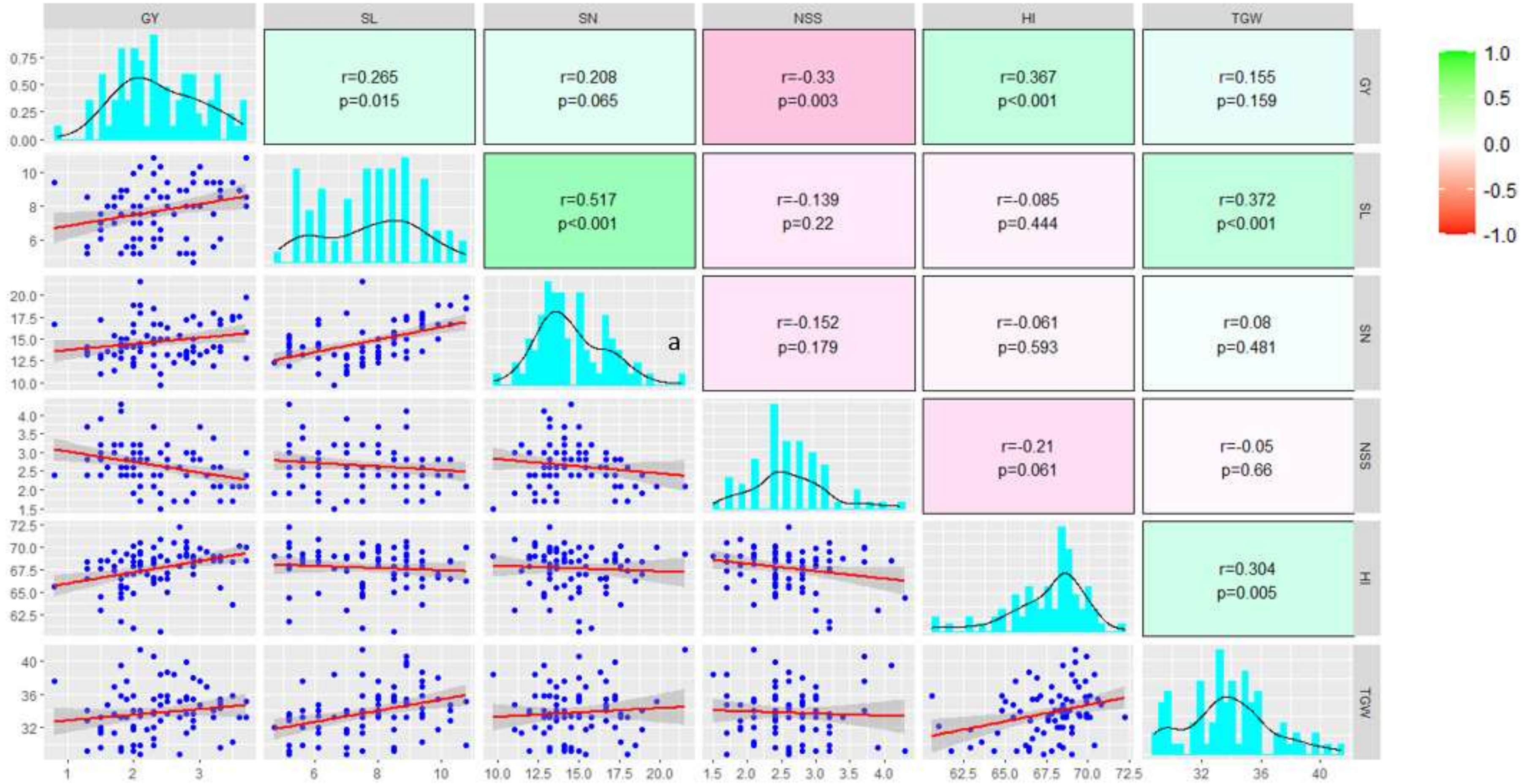

Fig. S1a Pearson's correlations among traits in LR the drought season of 2018

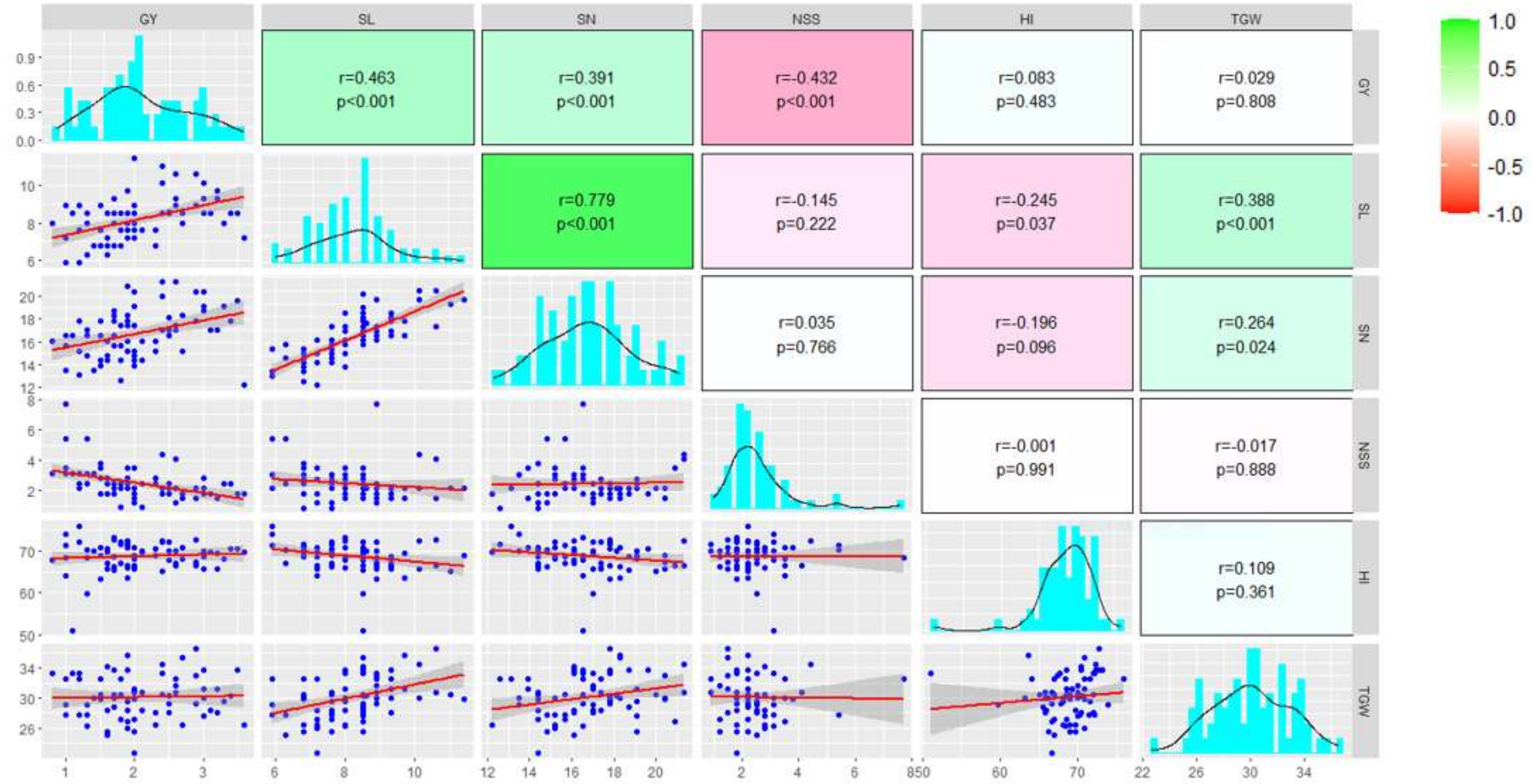

Fig. S1b Pearson's correlations among traits in MV in the drought season of 2018

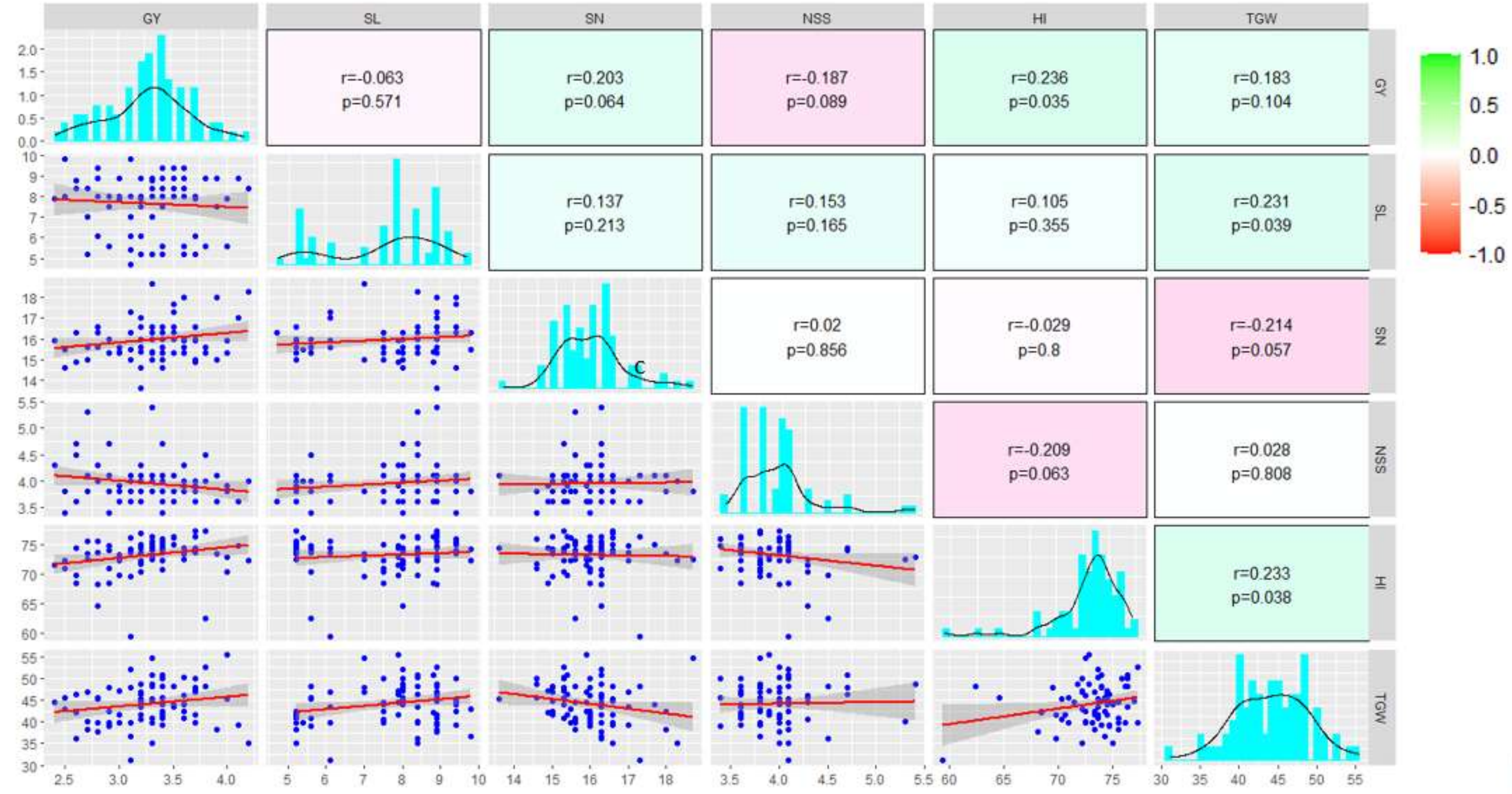

Fig. S1c Pearson's correlations among traits in LR in the favorable season of 2019

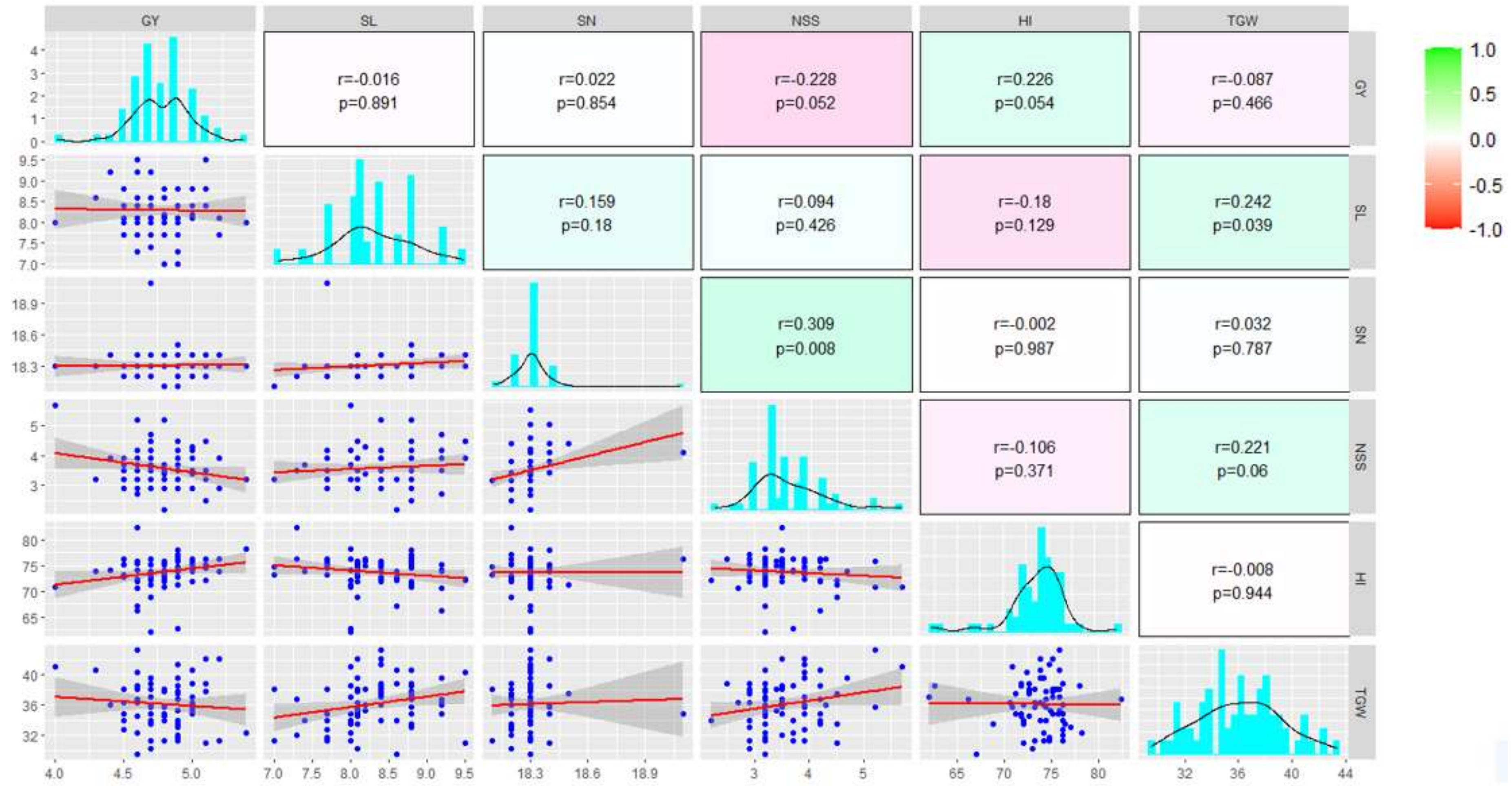

Fig. S1d Pearson's correlations among traits in MV in the favorable season of 2019

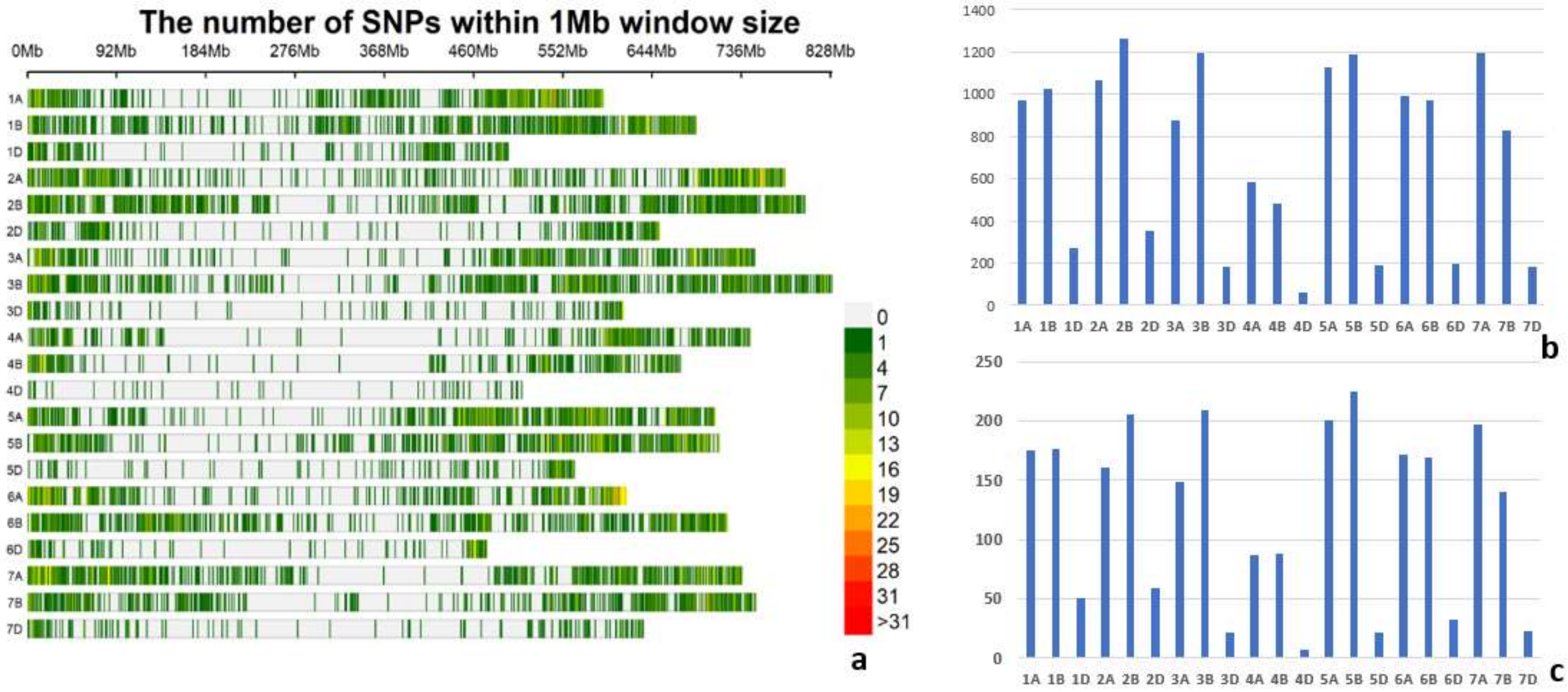

Fig. S2 Genome wide distribution of 15,208 SNPs (a, b) and 2,568 haplotype blocks (c) in the panel

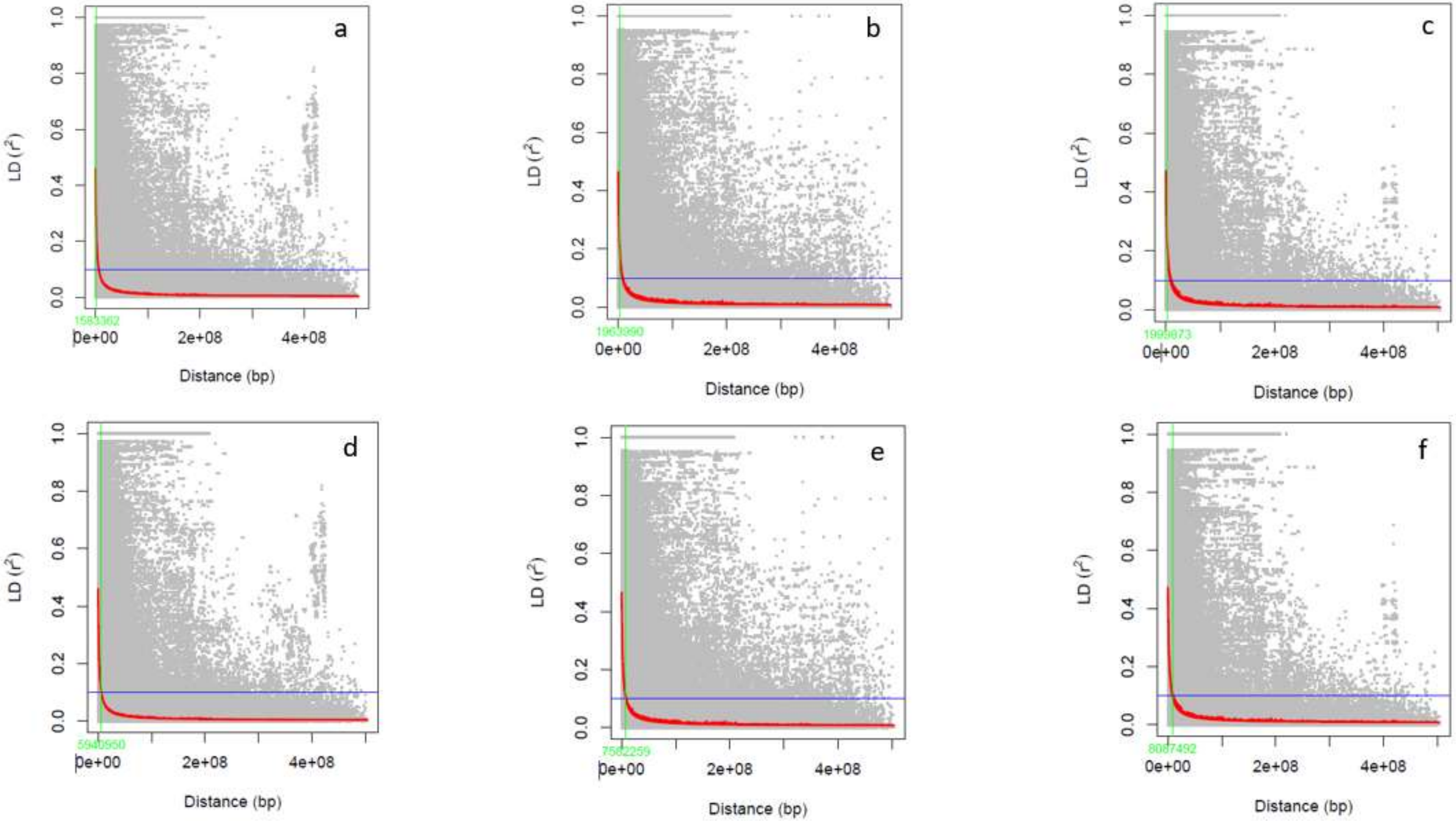

Fig. S3 Scatterplot showing LD decay estimated by plotting (r^2) against physical distance (bp). LD decay to half of its maximum value (a-c) and at cut off r^2 = 0.1 in the whole panel, landraces and modern varieties (d-f).

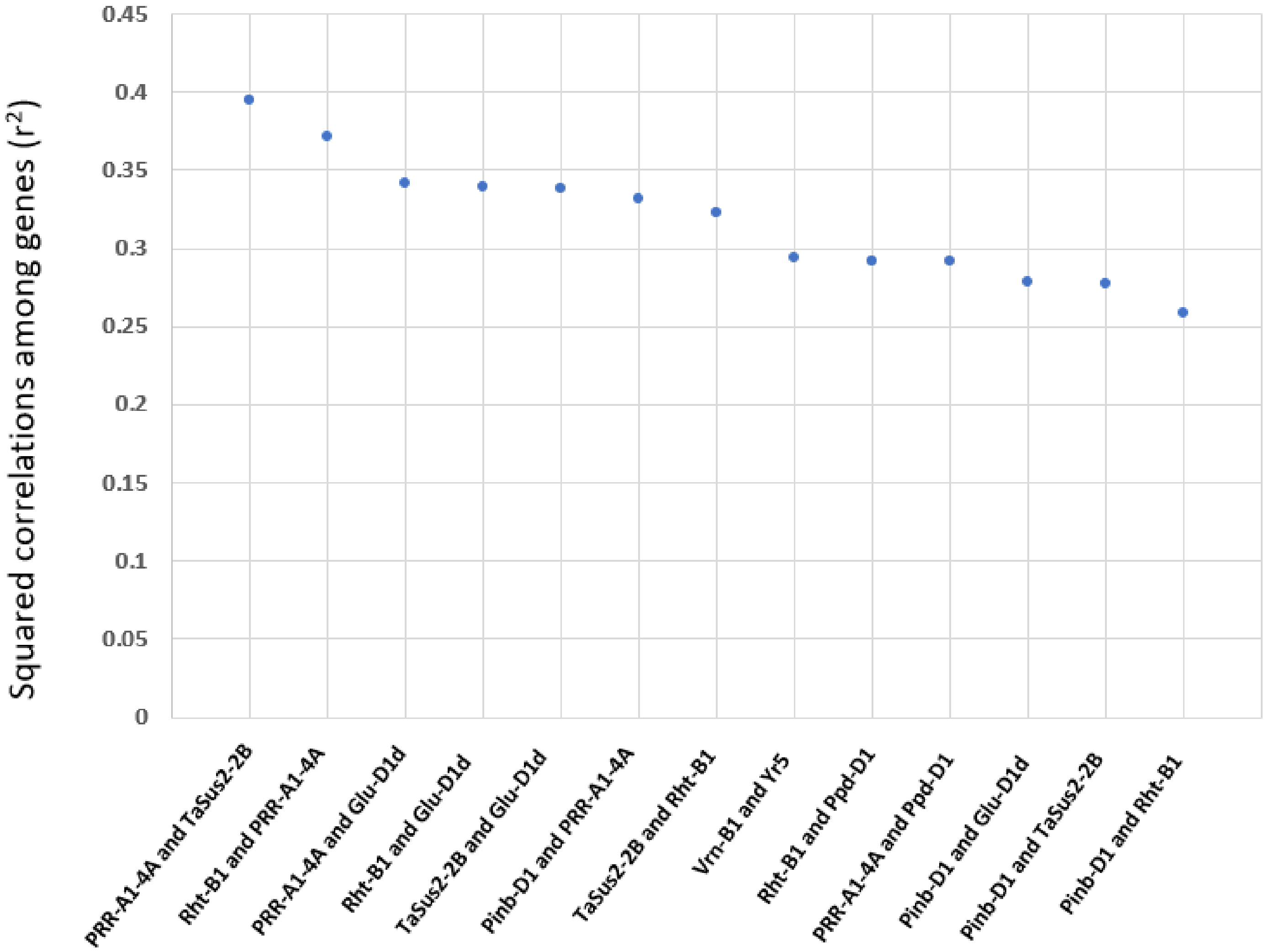

Fig. S4 Pairwise linkage disequilibrium values between genes that showed LD (r^2) > 0.25

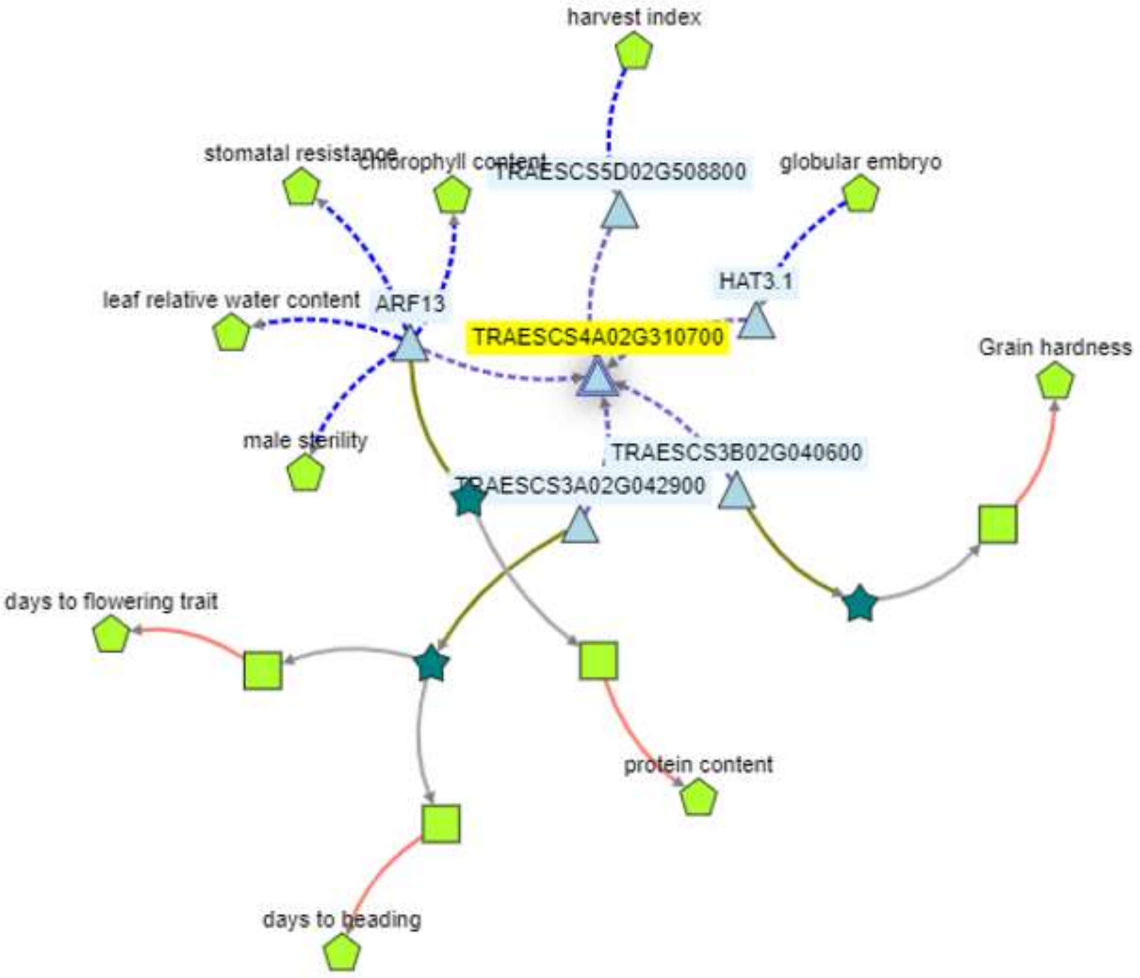

Fig. S5 Gene network and trait relationship analysis of candidate gene TraesCS4A02G310700 underlying H4A-48

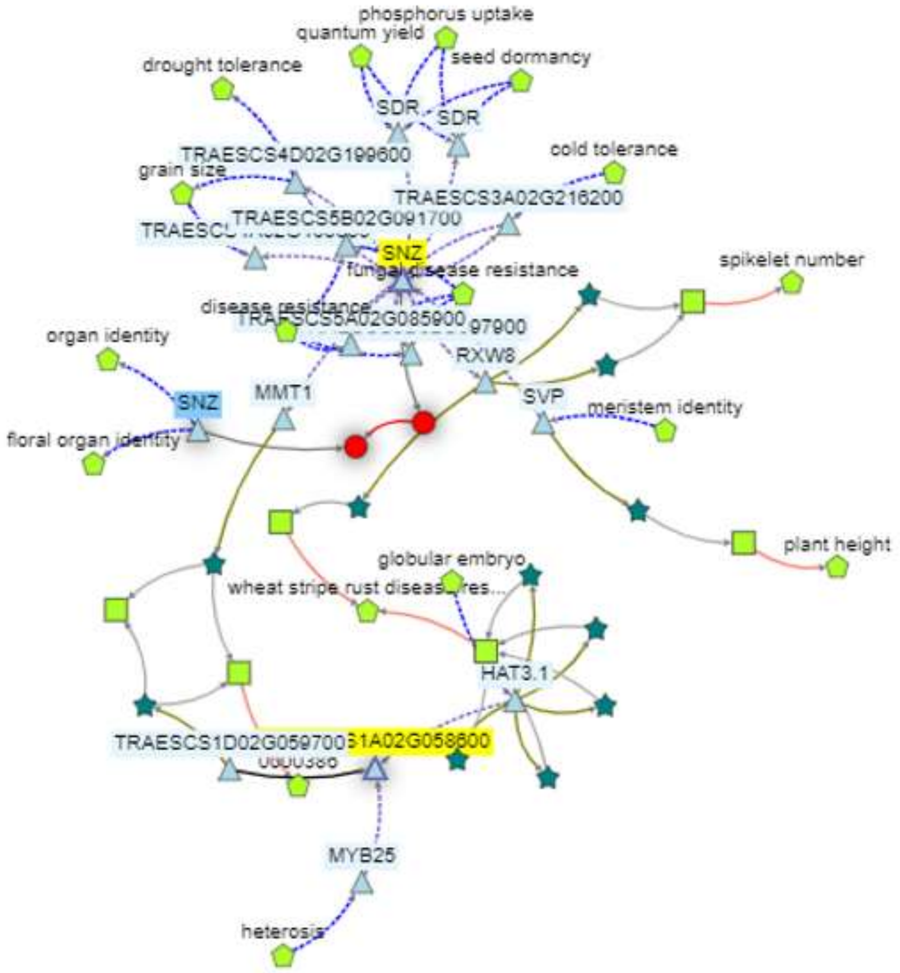

Fig. S6 Gene network and trait relationship analysis of candidate genes TraesCS1A02G058400 and TraesCS1A02G058600 underlying H1A-42
